# Structural and mechanistic aspects influencing the ADAM10-mediated shedding of the prion protein

**DOI:** 10.1186/s13024-018-0248-6

**Published:** 2018-04-06

**Authors:** Luise Linsenmeier, Behnam Mohammadi, Sebastian Wetzel, Berta Puig, Walker S. Jackson, Alexander Hartmann, Keiji Uchiyama, Suehiro Sakaguchi, Kristina Endres, Jörg Tatzelt, Paul Saftig, Markus Glatzel, Hermann C. Altmeppen

**Affiliations:** 1 Institute of Neuropathology, University Medical Center Hamburg-Eppendorf (UKE), Hamburg, Germany; 2 Institute of Biochemistry, Christian Albrechts University, Kiel, Germany; 3 German Center for Neurodegenerative Diseases (DZNE), Bonn, Germany; 4 Division of Molecular Neurobiology, Institute of Enzyme Research, Tokushima University, Tokushima, Japan; 5 Department of Psychiatry and Psychotherapy, University Medical Center, Johannes Gutenberg University, Mainz, Germany; 6 Institute of Biochemistry and Pathobiochemistry, Ruhr University, Bochum, Germany

**Keywords:** ADAM10, Antibody, Exosomes, Glycosylation, Membrane anchor, Neurodegeneration, Prion protein, Proteolytic cleavage, Shedding

## Abstract

**Background:**

Proteolytic processing of the prion protein (PrP^C^) by endogenous proteases generates bioactive membrane-bound and soluble fragments which may help to explain the pleiotropic roles of this protein in the nervous system and in brain diseases. Shedding of almost full-length PrP^C^ into the extracellular space by the metalloprotease ADAM10 is of peculiar relevance since soluble PrP stimulates axonal outgrowth and is protective in neurodegenerative conditions such as Alzheimer’s and prion disease. However, molecular determinates and mechanisms regulating the shedding of PrP are entirely unknown.

**Methods:**

We produced an antibody recognizing the neo-epitope of shed PrP generated by ADAM10 in biological samples and used it to study structural and mechanistic aspects affecting the shedding. For this, we investigated genetically modified cellular and murine models by biochemical and morphological approaches.

**Results:**

We show that the novel antibody specifically detects shed PrP in cell culture supernatants and murine brain. We demonstrate that ADAM10 is the exclusive sheddase of PrP^C^ in the nervous system and reveal that the glycosylation state and type of membrane-anchorage of PrP^C^ severely affect its shedding. Furthermore, we provide evidence that PrP shedding can be modulated by pharmacological inhibition and stimulation and present data suggesting that shedding is a relevant part of a compensatory network ensuring PrP^C^ homeostasis of the cell.

**Conclusions:**

With the new antibody, our study introduces a new tool to reliably investigate PrP-shedding. In addition, this study provides novel and important insight into the regulation of this cleavage event, which is likely to be relevant for diagnostic and therapeutic approaches even beyond neurodegeneration.

## Background

Proteolytic processing is an essential regulator of protein function and differs from many other posttranslational modifications by its irreversible character. As exemplified decades ago in the case of prohormones (such as the proopiomelanocortin [1]), differential or subsequent cleavages by endogenous proteases produce fragments with intrinsic biological functions, differing from the ones of the larger precursors. This concept may also help in understanding and explaining the biology of other “multifunctional” proteins, i.e. proteins with more than just one particular function ascribed to them.

One of these proteins is the cellular prion protein (PrP^C^), for which a multitude of physiological functions has been suggested in different tissues, cells and experimental settings [2, 3], even though not in each case without controversy or questionable reproducibility [4, 5]. For instance, PrP^C^ has been linked to developmental processes [6, 7], cell adhesion [8, 9], neurite outgrowth, axon guidance and synapse formation [10–14], as well as to neuroprotection [15–17] and regulation of the circadian rhythm [18]. Among the currently best characterized functions are its contributions to myelin maintenance [4, 19–21] and cellular homeostasis of divalent ions [22, 23] as well as its involvement in signaling events [24–26].

Too many functional implications for just one protein? Not necessarily. While transient interactions of PrP^C^ with alternating binding partners in different cellular locations may partially account for this functional diversity [5, 27], so might its proteolytic processing [28]. In fact, different highly conserved cleavage events occur constitutively on a relevant fraction of PrP^C^ [29–31], yet scientists are just starting to understand their biological relevance.

In contrast to some of the suggested physiological functions, the relevance of PrP^C^ in neurodegenerative proteinopathies is widely accepted. First and foremost, it is the essential substrate for the process of templated misfolding underlying fatal and transmissible prion diseases, such as Creutzfeldt-Jakob disease in humans or BSE in cattle [32–34]. Once having adopted its pathogenic conformation (PrP^Sc^), the prion protein is the key component of the infectious particles termed prions [32, 35–37]. Second, binding of toxic oligomeric protein species, such as PrP^Sc^ (in prion diseases [38]), Aβ (in Alzheimer’s disease [39–42]) or α-synuclein (in Parkinson’s disease [43, 44]), to PrP^C^ at the neuronal surface results in neurotoxic signaling. As for the physiological functions, increasing evidence suggests that proteolytic cleavages also impact on these pathogenic roles of the prion protein [28, 45, 46].

Here, we focus on the most membrane-proximate cleavage of PrP^C^, i.e. its shedding from the neuronal surface and release into the extracellular space by the metalloprotease ADAM10 [47, 48]. This cleavage not only regulates membrane levels of PrP^C^ and, thus, PrP^C^-related functions at the neuronal surface [28]. The resulting soluble fragment, shed PrP, likely has intrinsic functions as supported by studies using (recombinant) anchorless analogues, that showed beneficial effects with regard to axon outgrowth and synapse formation [13, 14] or neuroprotection [15, 49]. Focusing on neurodegeneration, we have recently shown a significant impact on the course of prion disease in mice by conditional depletion of the sheddase ADAM10 [50, 51], as have others by overexpression of exogenous ADAM10 [52] or by transgenic expression of anchorless versions of PrP [53, 54]. Moreover, by reducing membrane-bound PrP^C^ as a receptor and by producing anchorless PrP, which can block and detoxify Aβ and other harmful protein species in the extracellular space [55–58], shedding may also have a protective role in other, more frequent proteinopathies [45].

Surprisingly, shed PrP has recently been associated with the development of specific tumours in the nervous system, where it correlates with increased cancer cell proliferation [59]. In addition, a recent report shows critical involvement of shed PrP in the neuropathogenesis of HIV/AIDS by recruiting monocytes and aggravating the inflammatory response and the associated cognitive impairment [60].

Thus, given that shedding of PrP^C^ might provide a promising and potent target for therapy of various pathological conditions, a deeper mechanistic understanding and knowledge of factors influencing this cleavage is required. Here, we first introduce and characterize a novel antibody detecting shed PrP with high specificity and sensitivity. Using this tool, we investigate different structural (i.e. glycosylation state and membrane anchorage) and mechanistic aspects in vitro and in vivo for how they impact on this relevant proteolytic event. Finally, we show that shedding is part of a compensatory cellular network regulating PrP^C^ homeostasis.

## Methods

### Plasmids

The following constructs were used for transient transfection of cells. Detailed descriptions of the constructs can be found in the corresponding references: PrP-WT, PrP^C^ glycomutants PrP-G1, PrP-G2, PrP-G3 and anchor-mutant PrP^GPI-Thy1^ [61], PrP-TM (PrP-CD4 [62, 63]). All PrP constructs contained the 3F4 tag [64]. The N-terminally truncated PrP-C1 construct was cloned from the plasmid pcDNA3.1(+)/Zeo containing the murine *Prnp* gene. The sequence coding for the N-terminal part of PrP^C^ (aa23–110) was deleted by use of the restriction enzymes XbaI and HindIII and the resulting construct (Δaa23–110; i.e. PrP-C1) was verified by DNA sequencing.

### Antibody production

**Fig. 1:**
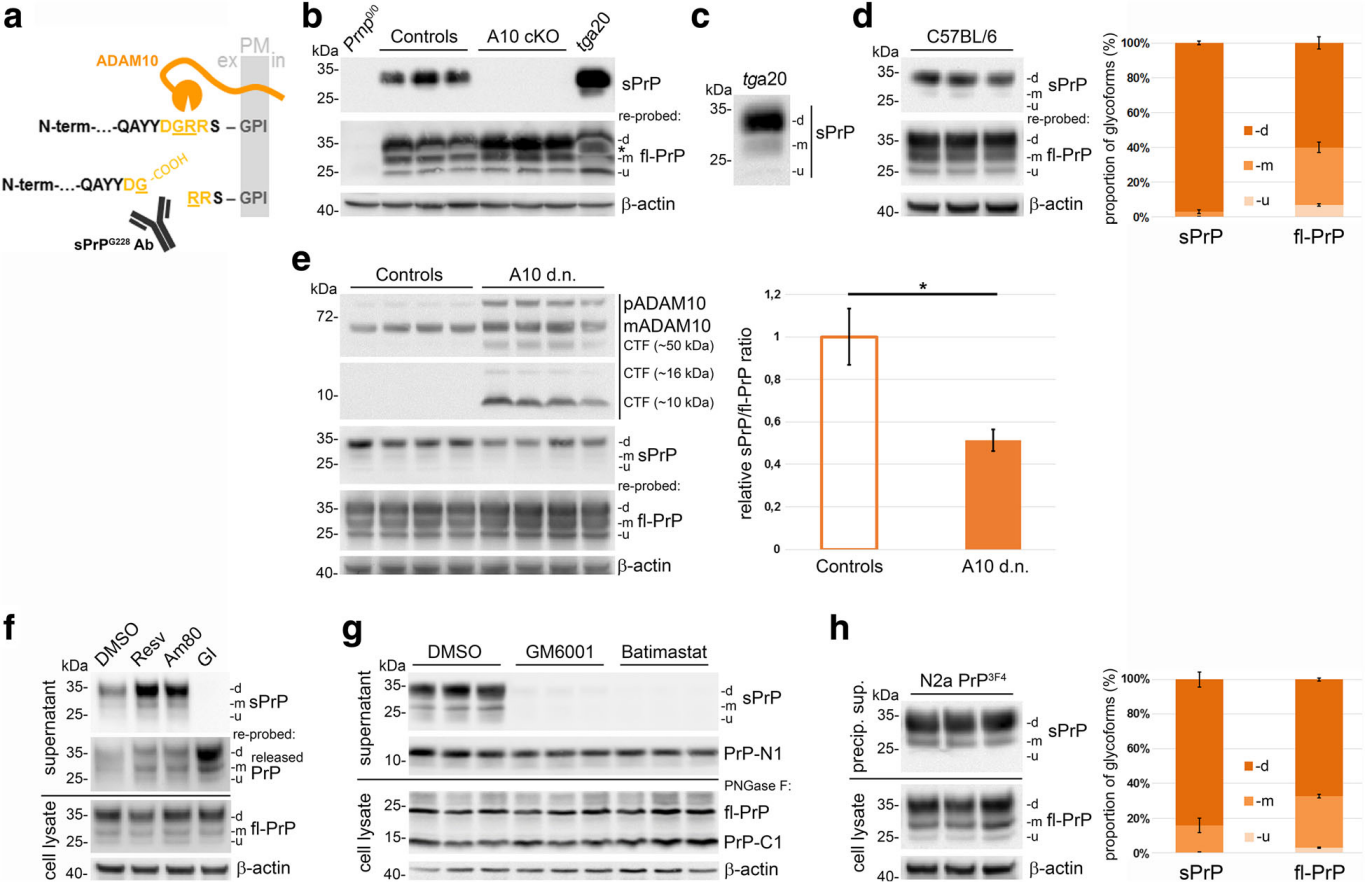
A new antibody directed against shed PrP^C^ reveals important aspects of the ADAM10-mediated cleavage. **a** Shedding of murine PrP^C^ by ADAM10 at the plasma membrane (PM). The sPrP^G228^ Ab is directed against the C-terminus Gly228 exposed after release of shed PrP into the extracellular space (ex). **b** Representative western blot of forebrain homogenates from *Prnp*^0/0^, wild-type control, ADAM10 cKO and *tg*a20 mice, first probed with sPrP^G228^ Ab and re-probed with POM2 against fl-PrP. An asterisk indicates position of signal from the initial detection of sPrP (in controls and *tg*a20 resulting in overexposure and blurred appearance when re-probed with POM2) and demonstrates a small shift in molecular weight between sPrP and fl-PrP. **c** Western blot of *tg*a20 brain shows that all PrP^C^ glycoforms can be shed, though a clear preference exists for the diglycosylated form. **d** Glycopattern analysis in three C57BL/6 mouse forebrains and quantification of glycoform proportions using sPrP^G228^ Ab and POM1 reveals a shift towards the diglycosylated form in sPrP compared to fl-PrP. **e** Western blot showing inhibitory effects of dominant-negative ADAM10 in forebrains of transgenic mice (A10 d.n.) compared to controls on PrP^C^ shedding. Premature/mature ADAM10 and C-terminal fragments (CTF) confirm genotypes. Quantification shows relative levels of sPrP referred to fl-PrP signal (controls set to one; *n* = 4; *p* = 0.014). **f**, **g** Representative analysis of precipitated supernatants and cell lysates of N2a cells treated with resveratrol (Resv), tamibarotene (Am80) or GI254023X (GI), GM6001 and Batimastat. Resveratrol and Am80 increased shedding (**f**), whereas the ADAM10-specific inhibitor GI (**f**) and metalloprotease inhibitors GM6001 and Batimastat (**g**) completely abolished it. **f** Re-probing the supernatant blot with POM2 shows an increase in fl-PrP released by other mechanisms. **g** No obvious effect on fl-PrP (here deglycosylated (PNGase F)) or N1/C1 levels was detected upon treatment. **h** Glycopattern comparison between sPrP (supernatants) and fl-PrP (lysates; *n* = 3) reveals predominance of shed diglycosylated PrP in N2a cells similar to findings in mice (**d**). β-actin = loading control; “d, m, u” = di-, mono-, unglycosylated PrP

The sPrP^G228^ antibody for the specific detection of shed PrP was generated by use of an anti-peptide approach and the classical 87-day polyclonal protocol (Eurogentec, Belgium). Briefly, based on the sequence information of murine PrP^C^ and previous determination of the cleavage site for ADAM10 [47], a recombinant peptide NH_2_-C-QAYYD**G**-COOH (in which **G**-COOH represents G228 as the new C-terminus of shed PrP exposed after cleavage (Fig. 1a)) was produced and N-terminally coupled to *Megathura crenulata* keyhole limpet hemocyanin (KLH) as carrier protein. This peptide was used as immunogen and injected into rabbit at days 0, 14, 28 and 56 of the programme. Bleedings were done at days 0, 38 and 66 to investigate the success of the immunization process by standardized ELISA tests. Animals were sacrificed and final bleeds were obtained at day 87. Standardized quality measures and affinity purification were performed at Eurogentec. Importantly, a second peptide (NH_2_-C-KESQAYYD**G**RRS-COOH) mimicking the C-terminus of fl-PrP (without the GPI anchor) was produced, coupled to a resin and served as a “negative control” to eliminate all antibodies from the polyclonal serum that would otherwise bind to fl-PrP.

### Rodent brain samples

Use of animal material in this study was in strict compliance with the Guide for the Care and Use of Laboratory Animals and ethics guidelines of the responsible local authorities. Frozen forebrain samples from wild-type C57BL/6, prion protein deficient (*Prnp*^0/0^ [65]), prion protein overexpressing (*tg*a20 [66]) mice as well as from mice with conditional knockout of ADAM10 in forebrain neurons (A10 cKO and wild-type littermate controls [67]), with transgenic overexpression of dominant negative ADAM10 (A10 d.n. and wild-type controls [68]), with depletion of sortilin-1 (Sort1 KO and wild-type controls [69]) or with a knock-in of 3F4-tagged PrP^C^ (PrP^3F4^KI and controls; both had a 192S4 background [70]), and from a rat and a rabbit were used to prepare 10% (*w*/*v*) homogenates in RIPA buffer (50 mM Tris-HCl pH 8, 150 mM NaCl, 1% NP-40, 0.5% Na-Deoxycholate, 0.1% SDS) freshly supplemented with Complete EDTA-free protease (PI) and PhosStop phosphatase inhibitor cocktails (Roche) on ice. Samples were homogenized with 30 strokes using a dounce homogenizer and incubated on ice for 20 min, shortly vortexed and incubated for another 20 min before centrifugation at 12,000 g at 4 °C for 12 min. Total protein content was assessed by Bradford assay (BioRad). Supernatants were either further processed for SDS-PAGE or stored at − 80 °C.

### Cell culture, transfection and treatments

Murine neuroblastoma cells (N2a) and mouse embryonic fibroblasts (MEF; [71]) were maintained at 37 °C under an atmosphere of 5% CO_2_ in Dulbecco’s modified Eagle’s medium (DMEM; Thermo Fisher Scientific) supplemented with 10% fetal bovine serum (FBS; Thermo Scientific Fisher). N2a PrP-KO cells were generated using the TALEN approach and characterized in detail before [72]. N2a PrP-KO cells were transfected using Lipofectamine 2000 (Thermo Fisher Scientific) following the manufacturer’s instructions. For stable overexpression of PrP^3F4^ in N2a PrP-KO (used for the glycopattern analysis shown in Fig. 1h) cells were kept for 3 weeks in selection media (Zeocin 400 μg/ml; Thermo Fisher Scientific) and single resistant clones were selected for amplification.

Treatments of cells were performed by adding the following compounds (and concentrations) to the cell culture media: Resveratrol (20 μM), Tamibarotene/Am80 (1 μM), GI254023X (3 μM), Tunicamycin (2.5 μg/ml), Swainsonine (5 μg/ml), Leupeptin (200 μg/ml). All compounds were purchased from Merck. These treatments were carried out in 6-well plates with 1 ml OptiMEM for 18 h overnight. In the case of Tunicamycin and Swainsonine cells were pretreated for 8 h. Treatment with GM6001 (25 μM) or Batimastat (10 μM) was for 10 h.

### Treatment of cells with PI-PLC

Two days post-transfection cells (grown in 6-well plate format) were incubated with 0.5 U/ml Phospholipase C (PI-PLC; Sigma-Aldrich) in 1 ml OptiMEM for 2 h at 37 °C, 5% CO_2_ in order to cleave GPI-anchor structures and release GPI-anchored proteins from the cellular surface. Supernatants were subsequently harvested and further processed while cells were lysed as described below.

### PNGase F and Endo H digestion

For removal as well as for investigations on processing and maturation of N-linked glycans attached to PrP^C^, cell lysates and/or supernatants were digested with either PNGase F or Endo H (New England Biolabs) according to the manufacturer’s protocols.

### Sample preparation, TCA precipitation, cell surface biotinylation assay, SDS-PAGE and western blot analysis

N2a cells were washed with PBS and lysed with RIPA buffer, incubated on ice for 15 min before centrifugation at 12,000 g for 12 min at 4 °C. The protein content of the resulting supernatant was determined by Bradford assay. Prior to SDS-PAGE, cell lysates or brain homogenates (see above) were mixed with 4× loading buffer (including β-mercaptoethanol) and denatured for 6 min at 96 °C.

For the analysis of cell culture supernatants, experiments were carried out with serum-free media (OptiMEM). Supernatants were precipitated with trichloroacetic acid (TCA). For this, supernatants were collected and immediately incubated with already dissolved protease inhibitor cocktail, cleared from dead cells and debris by mild centrifugations at 500 g and 5.000 g for 5 min each. 1/100 volume of 2% sodium deoxycholate (NaDOC) was then added and each sample was shortly vortexed. After 30 min incubation on ice, samples were mixed with 1/10 volume of 100% TCA and again incubated for 30 min on ice. After centrifugation at 15,000 g for 15 min at 4 °C, the supernatant was aspirated, and the air-dried pellet was dissolved in 1× loading buffer and boiled for 6 min at 96 °C.

For labelling and purification of proteins at the cell surface, a surface biotinylation assay was performed as described earlier [50] prior to cell lysis.

For SDS-PAGE, denatured samples were loaded on either precast Nu-PAGE 4–12% Bis-Tris protein gels (Thermo Fisher Scientific) or self-made 10% or 12% SDS-gels. After electrophoretic separation, proteins were transferred to nitrocellulose membranes (BioRad) by wet-blotting and membranes were subsequently blocked for 1 h with 5% skimmed dry milk dissolved in TBS-T (containing 0.1% Tween-20) and incubated with primary antibody diluted in 5% skimmed dry milk in TBS-T overnight at 4 °C on a shaking platform. For detection of full length PrP^C^ (fl-PrP), mouse monoclonal antibodies POM1 (1 μg/ml), POM2 (0.6 μg/ml) [73] or, in the case of the sortilin-1 knockout mouse brains (Fig. 5j), SAF61 (0.2 μg/ml; Bertin Pharma) were used. Proteolytically shed PrP^C^ was detected with our new rabbit polyclonal sPrP^G228^ antibody (0.2 μg/ml) characterized in detail herein. Moreover, we used anti-ADAM10 (0.4 μg/ml; abcam), anti-mouse β-amyloid antibody for detection of sAPPα (1 μg/ml; BioLegend), anti-actin antibody clone C4 (MAB1501, 1:1000; Merck) and anti-Flotillin-1 clone 18 (0.25 μg/ml; BD Biosciences). Membranes were subsequently washed with TBS-T and incubated for 1 h with respective HRP-conjugated secondary antibodies and subsequently washed 6× with TBS-T. After incubation with Pierce ECL Pico or Super Signal West Femto substrate (Thermo Fisher Scientific), chemiluminescence was detected with a ChemiDoc imaging station (BioRad) and densitometrically quantified using Image Studio Lite software version 5.2 (LI-COR).

### Immunofluorescence staining of surface proteins and microscopy

N2a cells were grown on glass coverslips. After washing with PBS, living cells were incubated for 20 min on ice (to avoid endocytosis) with the primary antibody dissolved in 2% BSA/PBS. Surface PrP^C^ was detected with POM1 antibody (10 μg/ml). After several washes with PBS, cells were incubated with suitable secondary antibodies for 20 min on ice, subsequently fixed in 4% paraformaldehyde for 20 min at room temperature and mounted on glass slides with DAPI Fluoromount G (Southern Biotech). Analysis was performed using a TCS SP5 confocal microscope (Leica).

### Histological and immunohistochemical stainings

Sampling, formalin fixation, paraffin embedding, hematoxilin and eosin (H&E) staining as well as immunostaining with anti-prion protein antibody SAF84 (Caiman Chemical) of murine brain samples has been described earlier [50]. Immunostaining of shed PrP was likewise performed in one run using a Benchmark XT machine (Ventana) to allow for best comparability. In brief, deparaffinated brain sections were boiled for 1 h in citrate buffer (CC1 Cell Conditioning Solution, Ventana) for antigen retrieval and then incubated for 1 h with the sPrP^G228^ primary antibody (7 μg/ml; in antibody diluent solution (Zytomed) with 5% goat serum). Detection with anti-rabbit secondary antibody (Nichirei Biosciences) and Ultra View Universal DAB Detection kit (Ventana), as well as blue counterstaining were performed by the machine following standardized protocols.

### Exosome isolation and nanoparticle tracking analysis

N2a cells were cultured in OptiMEM for 18 h. For the harvest of extracellular vesicles (here further referred to as exosomes), cell culture supernatants were first complemented with PI and centrifuged for 10 min at 1000 g and further at 7500 g for 15 min at 4 °C, followed by filtration through a 22 μm membrane to clear it from dead cells and debris. Exosomes were then pelleted by ultracentrifugation at 100,000 g for 70 min at 4 °C in an Optima L-100 XP using a SW40Ti rotor (Beckman Coulter, Inc.) and subsequently resuspended in PBS containing PI. For quantification and characterization, a NanoSight LM14 (Malvern Instruments) equipped with a 638 nm laser and a Marlin F033B IRF camera (Allied Vision Technologies) was used. For each sample, 10 videos of 10 s length were recorded with a camera intensity setting of 16 and analysed to assess average size and concentration of exosomes using the batch processing function of the software. For normalized western blot analysis, 5 × 10^10^ exosomes per sample were used.

### Statistical analysis

For experiments using mouse brain samples, *n* refers to the number of biological samples (i.e. mice) per experimental group. For cell culture-based data, *n* stands for the number of independent experiments. Statistical comparison of western blot quantifications was performed using Student’s t-test and significance was considered with *p*-values as follows: *p < 0.05, **p < 0.005, ***p < 0.001.

## Results

### A novel antibody specifically detects shed PrP and reveals important insight into the ADAM10-mediated shedding of PrP^C^ in mice and cells

ADAM10 is the relevant sheddase of PrP^C^ releasing a soluble form (shed PrP, sPrP) from the plasma membrane [47, 48]. Since membrane-bound full length (fl) PrP^C^ and its shed form only differ in three amino acids (murine sequence) and the GPI-anchor, it is hard to reliably discriminate between both in most approaches. Based on available sequence information and the previous identification of the cleavage site for ADAM10 [47], we therefore generated an antibody specific for sPrP (sPrP^G228^) being directed against the newly generated carboxy-terminus at Glycine 228 (G228) exposed after cleavage (Fig. 1a).

To characterize this antibody in detail, we analyzed forebrain homogenates of age-matched *Prnp*^0/0^ mice (as negative control), recently described mice with neuron-specific (CamKIIα-driven) depletion of ADAM10 (A10 cKO; to control for specificity) as well as wild-type littermate controls, and PrP^C^-overexpressing *tg*a20 mice (as positive controls) by western blot. As expected, detection with our new antibody consistently revealed no signal in *Prnp*^0/0^ samples, basal levels in wild-type mice and strongly increased signal intensity in *tg*a20 mice (Fig. 1b). Though we expected significantly reduced levels of shed PrP in A10 cKO mice, to our surprise we could not detect any signal in these samples. Besides supporting the specificity of the antibody, this indicates that no other cell types or proteases contribute to this cleavage in brain. Re-probing the same blot with an antibody against fl-PrP revealed an increase in total PrP^C^ levels in A10 cKO mouse brains (Fig. 1b), a finding that has been made earlier and can be attributed to the lack of shedding [50]. Moreover, while this blotting strategy demonstrated the overlapping banding pattern (as well as the masking of sPrP signals by excess amounts of fl-PrP using common PrP antibodies), it also revealed a small shift in the molecular weight of sPrP corresponding to the lack of three amino acids and the GPI-anchor (Fig. 1b and Additional file 1). We also investigated the species specificity of the new antibody using mouse, rat and rabbit brain samples. As expected for the different C-terminal PrP^C^ sequences, the sPrP^G228^ antibody only detected sPrP in brain homogenates of mice and rat (Additional file 2).

Though not being in the focus of this study, we were also interested in the applicability of the antibody in morphological analyses and performed immunohistochemical staining of paraffin-embedded mouse brain sections. As exemplified for the hippocampal area in Additional file 3, no signal was obtained in a *Prnp*^0/0^ mouse, whereas a diffuse staining was found in wild-type and, with higher intensity, in *tg*a20 brain as could be expected for a soluble fragment distributed in the brain parenchyma.

Although, structurally, all three glycoforms of PrP^C^ can be shed (as demonstrated in *tg*a20 brain (Fig. 1c)), a strong predominance of the diglycosylated form of sPrP was obvious in all of our biochemical analyses. To investigate this in more detail, we analyzed the glycopattern of sPrP compared to cell-associated fl-PrP in brain homogenates of wild-type mice (Fig. 1d) and found a clear shift and a drastic increase in the proportion of diglycosylated sPrP (mean: 97 ± 1%; compared to 60 ± 4% for fl-PrP; *n* = 3; ±SD) with only little mono- (3 ± 1%; fl-PrP: 33 ± 3%) and almost no unglycosylated sPrP (0.07 ± 0.03%; fl-PrP: 6.8 ± 0.8%).

As a model for downregulation of ADAM10-mediated cleavage events, we investigated PrP shedding in forebrains of mice overexpressing a dominant negative form of ADAM10 (A10 d.n.) in addition to the endogenous protease [68] (Fig. 1e). When referring the sPrP to the respective fl-PrP signal, we found a ~ 50% reduction (mean sPrP/fl-PrP ratio: 0.51 ± 0.05; *n* = 4; ±SEM) in A10 d.n. mice compared to matched controls (set to 1.00 ± 0.13).

Since for main parts of this study we used N2a cells transfected with murine versions of PrP^C^ containing the 3F4 tag in the middle of the protein sequence, we first had to show that this modification does not influence the shedding event. This is even more important as it is known, that the course of prion diseases is altered by this tag [70, 74]. We therefore decided to investigate shedding in the best possible model, i.e. in PrP^3F4^ knock-in (KI) mice expressing levels of 3F4-tagged PrP identical to PrP^C^ levels in wild-type mice (Additional file 1) [70]. No significant differences in sPrP levels were observed between controls (set to 1.00 ± 0.23; n = 3; ±SD) and PrP^3F4^ KI mice (0.85 ± 0.12) thus ruling out an impact of this modification on PrP shedding as could be expected from its intramolecular distance to the membrane-proximate shedding site.

We next employed the new antibody in cell culture-based experiments. Given that manipulation of PrP^C^ shedding may become a therapeutic option in different pathologies, we investigated how pharmacological stimulation and inhibition of ADAM10 affect sPrP production in N2a cells (Fig. 1f,g). Among others, the stilbenoid resveratrol and the synthetic retinoid tamibarotene (Am80) have been successfully used to increase ADAM10-mediated cleavage events [75, 76]. We also found elevated levels of sPrP in supernatants of N2a cells treated with these substances compared to solvent-treated controls (Fig. 1f). In contrast, shedding was abolished upon treatment with the ADAM10-selective inhibitor GI254023X (GI) [77]. Of note, upon re-probing the “supernatant blot” with another PrP antibody (POM2), a strong signal was obtained under GI-treatment indicative of a release of fl-PrP by alternative routes when shedding is blocked (as discussed later). Fittingly, cell-associated PrP^C^ levels (in lysates) remained rather unaffected by the different treatments further supporting existence of compensatory mechanisms regulating PrP^C^ homeostasis in N2a cells (discussed later). We also assessed the metalloprotease inhibitors GM6001 and batimastat (Fig. 1g). These drugs likewise abolished the shedding of PrP^C^ at the cell surface yet did not significantly alter production of N1 and C1 fragments resulting from the α-cleavage of PrP^C^. There is controversy regarding the involvement of ADAMs in the α-cleavage (reviewed in [45, 78]). However, due to the lack of membrane permeability of the inhibitors used here, this finding cannot count as an argument against ADAMs as potential “α-PrPases”, given that α-cleavage is thought to occur mainly within the secretory pathway [79]. Again, levels of cell-associated fl-PrP did not appear to be altered by these treatments.

Lastly, consistent with our findings in mouse brain (Fig. 1d), we also observed a changed glycopattern of sPrP compared to fl-PrP in N2a cells (Fig. 1h; diglycosylated: 84.2 ± 4.4% (sPrP) vs. 67.4 ± 0.9% (fl-PrP); monoglycosylated: 15.6 ± 4.2% (sPrP) vs. 29.6 ± 1.0% (fl-PrP); unglycosylated: 0.22 ± 0.18% (sPrP) vs. 2.9 ± 0.3% (fl-PrP); *n* = 3; ±SD) though relatively more monoglycosylated sPrP is found in N2a cells (15.6 ± 4.2%) than in brain (3 ± 1%; Fig. 1d). To clarify whether our findings indicate a real preference for the shedding of diglycosylated PrP or rather reflect the availability of different glycoforms at the plasma membrane, we performed cell surface biotinylation and glycopattern analysis in N2a cells (Additional file 4). Though relatively more diglycosylated PrP is indeed available at the plasma membrane (compared to total PrP levels in cell lysates; Additional file 4B), our data still argues in favor of a preference for diglycosylated PrP given the strong predominance of this form among shed PrP (Fig. 1d, h). In summary, we have generated a sensitive and highly specific antibody to discriminate between shed and fl-PrP in mouse brains and cell culture supernatants. ADAM10 on neurons seems to be the dominant (if not exclusive) PrP sheddase. ADAM10-mediated shedding of PrP^C^ can be modulated by various means, and our shedding-specific antibody is a useful read-out tool for such experiments. Though all glycoforms can in principle be shed, diglycosylated PrP by far represents the major substrate for ADAM10.

### The glycosylation state impacts on PrP shedding

**Fig. 2:**
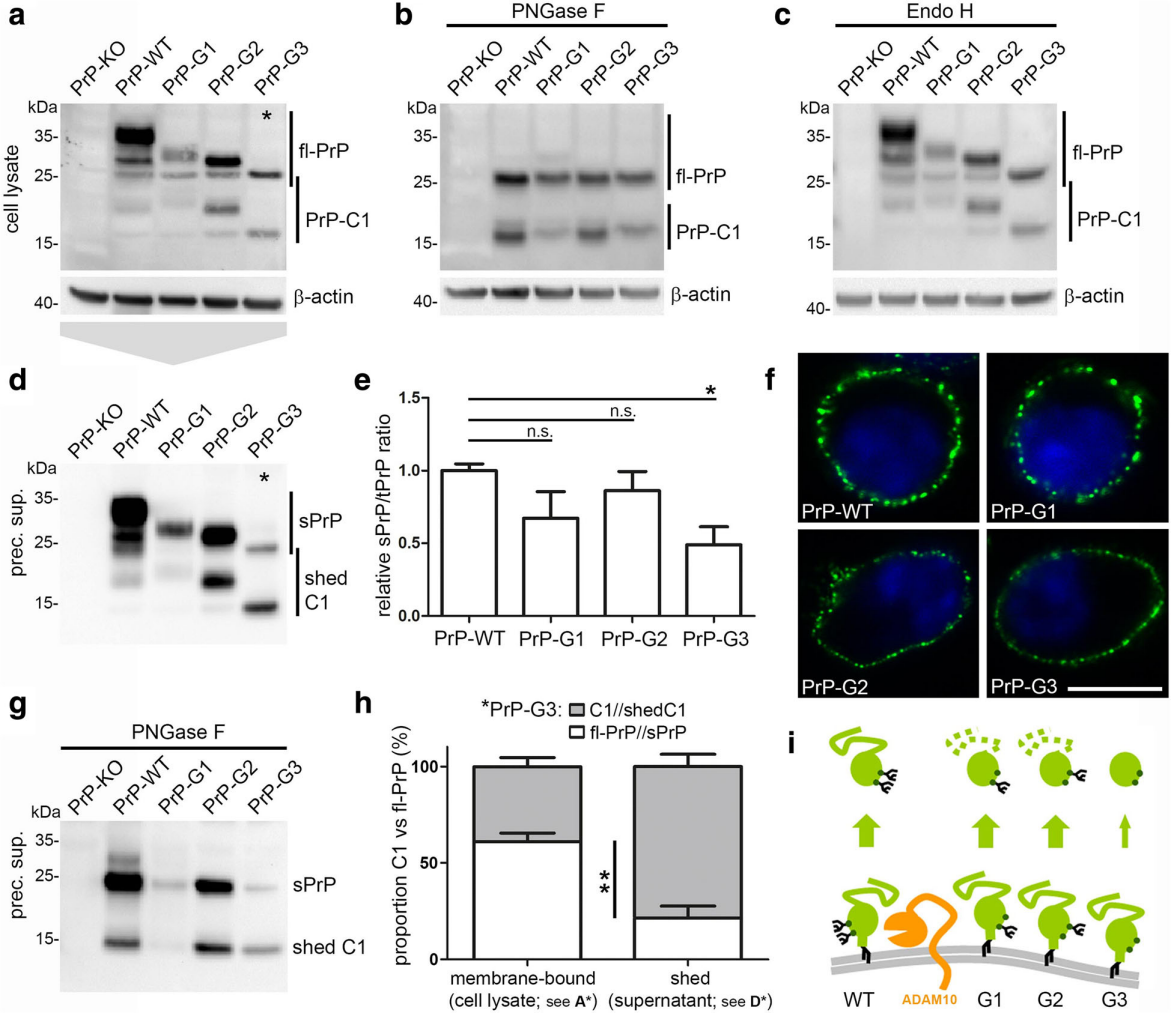
PrP glycosylation mutants are differentially shed. Representative western blot characterization of PrP^C^ depleted N2a cells (PrP-KO) transfected with wild-type PrP (PrP-WT) or the PrP glycomutants N180Q mutant (PrP-G1), N196Q (PrP-G2) and N180Q/N196Q (PrP-G3) showing the glycopattern in untreated lysates (**a**) as well as upon deglycosylation with PNGase F (**b**). **c** Digestion of lysates with Endo H does not reveal obvious alterations compared to the glycopattern in undigested samples (**a**), proving correct sorting and processing of the glycomutants. The differentially glycosylated C1 fragments resulting from α-cleavage of PrP^C^ are also detected in A,B and C. Actin was detected as loading control. **d** Analysis in precipitated media supernatant using the sPrP^G228^ Ab reveals that not only PrP but likewise its C1 fragments are shed in PrP-WT and all glycomutants. **e** Quantification of PrP shedding. For normalization, intensity of sPrP signals in media was referred to total amounts of PrP in cell lysates. A trend of reduced shedding is observed for all glycomutants when compared to PrP-WT and a significant decrease is found for unglycosylated PrP-G3 (*n* = 3; *p* = 0.018). **f** Confocal microscopy of PrP surface staining confirms presence of all glycomutants at the cell surface (scale bar = 10 μm). **g** PNGase F digestion of precipitated cell culture supernatant. **h** For the unglycosylated G3 mutant we found a significant shift in the C1 to PrP ratio between cell lysates (membrane-bound forms; see asterisk in **a**) and supernatants (shed forms; see asterisk in **b**). Intensity for PrP-C1 in supernatants or lysates was referred to the respective fl-PrP signal. Shed C1 is significantly increased compared to membrane-bound C1 (n = 3; *p* = 0.007). **i** Schematic representation of PrP shedding summarizing the reduced shedding and the relative preference for C1 in mutants with impaired glycosylation

Glycosylation of PrP^C^ impacts on its biology and role in prion disease [62, 80–83]. Given the predominance of diglycosylated sPrP under normal conditions (Fig. 1), we wondered how shedding would be affected if only specific glycoforms of PrP^C^ are present in cells. To this end, we transfected PrP^C^-depleted N2a cells (PrP-KO; generated using the TALEN strategy and described earlier [72]) with either wild-type PrP or PrP glycomutants carrying a mutation in either the first (N180Q mutant; PrP-G1), the second (N196Q mutant; PrP-G2) or both (N180Q/N196Q mutant; PrP-G3) N-glycosylation sites and, thus, giving rise to mono- (G1 and G2) or unglycosylated (G3) PrP^C^. Using these glycomutants, we could previously demonstrate a relevant impact of the N-glycans on the sorting of PrP^C^ in polarized cells [61]. Despite differences in transfection efficiencies, western blot analysis revealed the typical banding pattern for the glycomutants as observed for similar mutants in transgenic mice (Fig. 2a) [83]. Like fl-PrP, the membrane-attached C1 fragment resulting from α-cleavage of PrP^C^ also presents with a three-banding pattern with the diglycosylated C1 overlapping with unglycosylated fl-PrP. For a better characterization, deglycosylation was performed to only obtain unglycosylated fl-PrP and C1 fragment and showed that all mutants undergo α-cleavage (Fig. 2b). No effect was observed upon treatment of lysates with Endo H indicating a correct processing of the glycans and trafficking out of the ER and to the cellular surface for all transfected mutants (Fig. 2c). Immunofluorescence analysis of surface PrP^C^ further supported a correct biosynthesis and showed that all mutants are readily expressed at the plasma membrane (Fig. 2f) confirming previous results in polarized MDCK cells [61]. Of note, analysis of sPrP in media supernatants revealed that not only fl-PrP but also the truncated C1 fragment can be shed for all glycoforms (Fig. 2d; deglycosylation of supernatants for confirmation of bands is shown in Fig. 2g). Quantification (Fig. 2e) of sPrP referred to total PrP (to correct for different transfection efficiencies) showed a moderate decrease in sPrP for the monoglycosylated mutants, yet a significant reduction to ~ 50% was observed for the unglycosylated PrP-G3 (mean: 0.49 ± 0.11 compared to PrP-WT set to 1.00 ± 0.04; *n* = 3; ±SEM). Interestingly, as indicated by asterisks in Fig. 2a and d, for the unglycosylated PrP-G3 mutant we consistently detected a significant difference in the ratio of PrP and C1 between membrane-associated (fl-PrP: 61%; C1: 39%; ±4.5% SEM; n = 3) and shed forms (sPrP: 21%; shed C1: 79%; ±6.2%). This increase in the proportion of C1 (from 39% in lysates to 79% among the shed PrP forms) in the absence of N-glycans may relate to the longer half-life of C1 at the plasma membrane and a fast re-internalisation of unglycosylated fl-PrP [84–86] which seems to be disfavored as a substrate (Fig. 2i). In contrast, for normally glycosylated PrP-WT, there appeared to be a preference for the shedding of (diglycosylated) fl-PrP over the (diglycosylated) C1 fragment when comparing fl-PrP and C1 ratios in PNGase F digested lysates (Fig. 2b) and media supernatants (Fig. 2g). This observation prompted us to investigate a potential influence of the N-terminal half of PrP^C^ on the membrane-proximate shedding. We therefore transfected PrP-KO N2a cells with PrP-WT or with an N-terminally truncated construct (PrP-C1) corresponding to the physiological C1 fragment (Additional file 5). Despite indicating that a preference for the shedding of diglycosylated forms also exists for the C1 fragment, shedding of the latter was significantly reduced compared to fl-PrP in cells transfected with PrP-WT. Although further analysis are clearly required and differences might partially result from transient overexpression and altered surface expression of the constructs, these findings point to a role of the PrP N-terminal domain in the C-terminal shedding event. Thus, the glycosylation state as well as proteolytic truncation seem to affect PrP^C^ shedding.

### Shedding is also affected by pharmacological modulation of PrP^C^ glycosylation

**Fig. 3:**
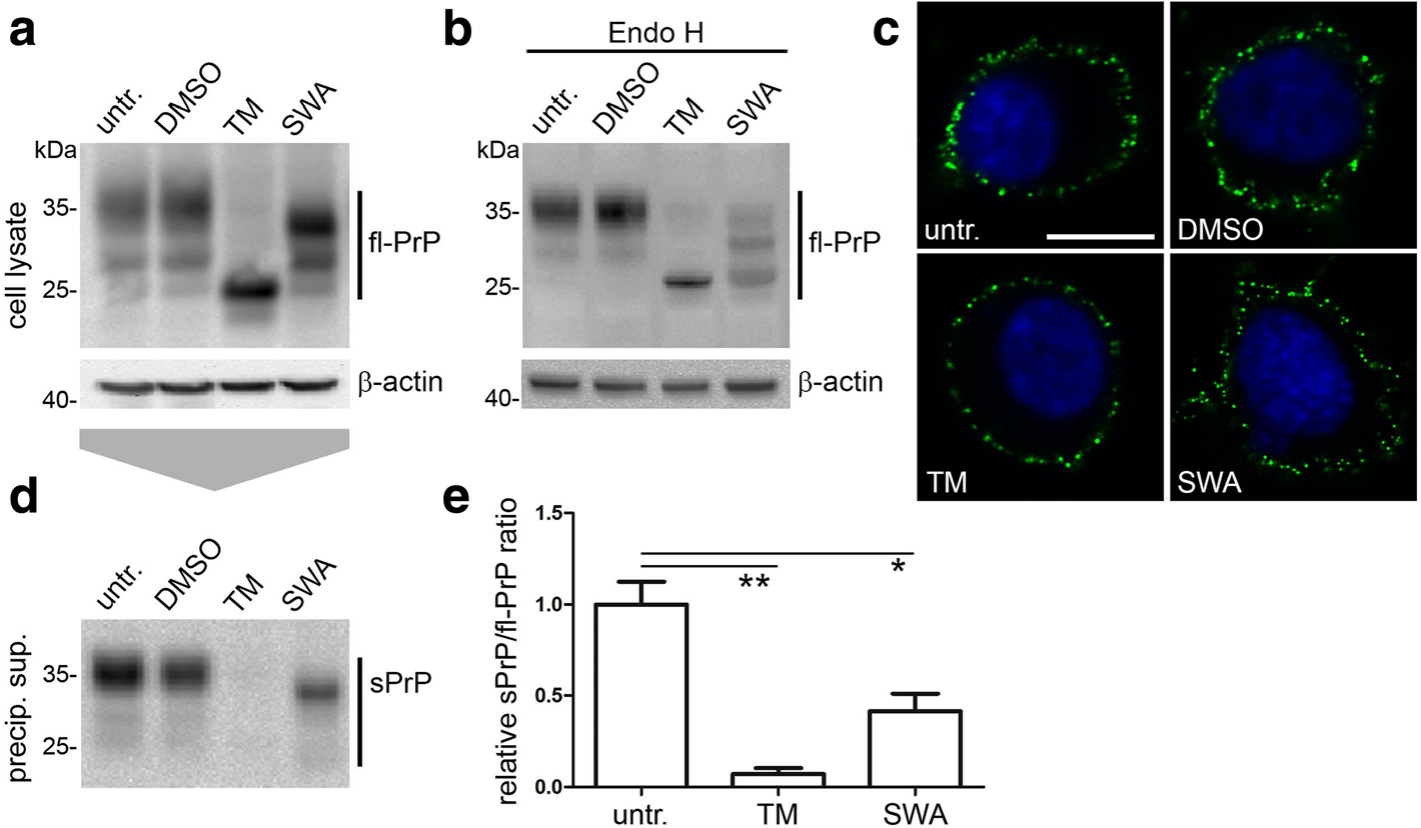
Pharmacological manipulation of N-glycosylation alters PrP shedding. **a** Representative western blot analysis of wild-type N2a cells either left untreated or treated with DMSO (as a solvent control), 2.5 μg/ml tunicamycin (TM) or 5 μg/ml swainsonine (SWA). For TM only unglycosylated PrP is detected. The SWA-treated sample reveals a slight downwards shift for diglycosylated PrP compared to DMSO- and untreated samples. **b** Endo H digestion further demonstrates an altered glycopattern for SWA-treated cells indicating that correct maturation to complex N-glycans was (at least partially) impaired. Actin served as loading control. **c** Staining on non-permeabilized cells demonstrating that, in all conditions, PrP^C^ is sufficiently expressed at the plasma membrane. **d** Representative western blot showing shed PrP in corresponding (precipitated) media supernatants. **e** Quantification of shedding efficiency (measured as the ratio of shed PrP in supernatants to fl-PrP in respective lysates) showing almost abolished shedding in TM-treated (n = 3; *p* = 0.002) and impaired shedding in SWA-treated cells (n = 3; *p* = 0.020)

To support our findings obtained with PrP glycomutants (Fig. 2), we pharmacologically manipulated glycosylation in wild-type N2a cells. While the antibiotic tunicamycin (TM) inhibits N-glycosylation, the alkaloid swainsonine (SWA) is a known inhibitor of the further maturation of N-linked glycan structures resulting in non-mature high-mannose glycans. Treatment of cells with TM completely prevented N-glycosylation of PrP^C^, whereas treatment with SWA resulted in a shift in the molecular weight of diglycosylated PrP^C^ indicating immature glycosylation (Fig. 3a). Digestion of lysates with Endo H revealed that SWA (partially) impaired complex glycosylation as shown by an altered glycopattern compared to controls (Fig. 3b). Further confirmation for the TM- and SWA-treatments and the enzymatic deglycosylation reactions is presented in a side-by-side comparison (Additional file 6). However, it should be noted that we did not reach a complete deglycosylation in the case of Endo H-digestion of lysates of SWA-treated cells. Presence of residual complex glycosylated PrP^C^ suggests that the incubation with SWA (8 h) was too short or that Endo H digestion was incomplete. Independent of the type of treatment, PrP^C^ was expressed at the cellular surface (Fig. 3c). Intriguingly, PrP^C^ shedding was almost completely abolished in cells treated with TM (mean: 0.07 ± 0.03; *n* = 3; SEM) and significantly reduced upon treatment with SWA (0.41 ± 0.10) compared to untreated controls (set to 1.00 ± 0.12) (Fig. 3d, e). Even though only little non-mature diglycosylated PrP^C^ was present in SWA-treated cells (Fig. 3b), this fraction seems to be the only relevant substrate for shedding. Again, these data support our previous findings (Figs. 1 and 2) showing that diglycosylated PrP is the preferential substrate for ADAM10 at the cell surface and that altered glycosylation influences PrP shedding efficiency.

### Membrane anchorage and topology of PrP^C^ determine its shedding efficiency

**Fig. 4:**
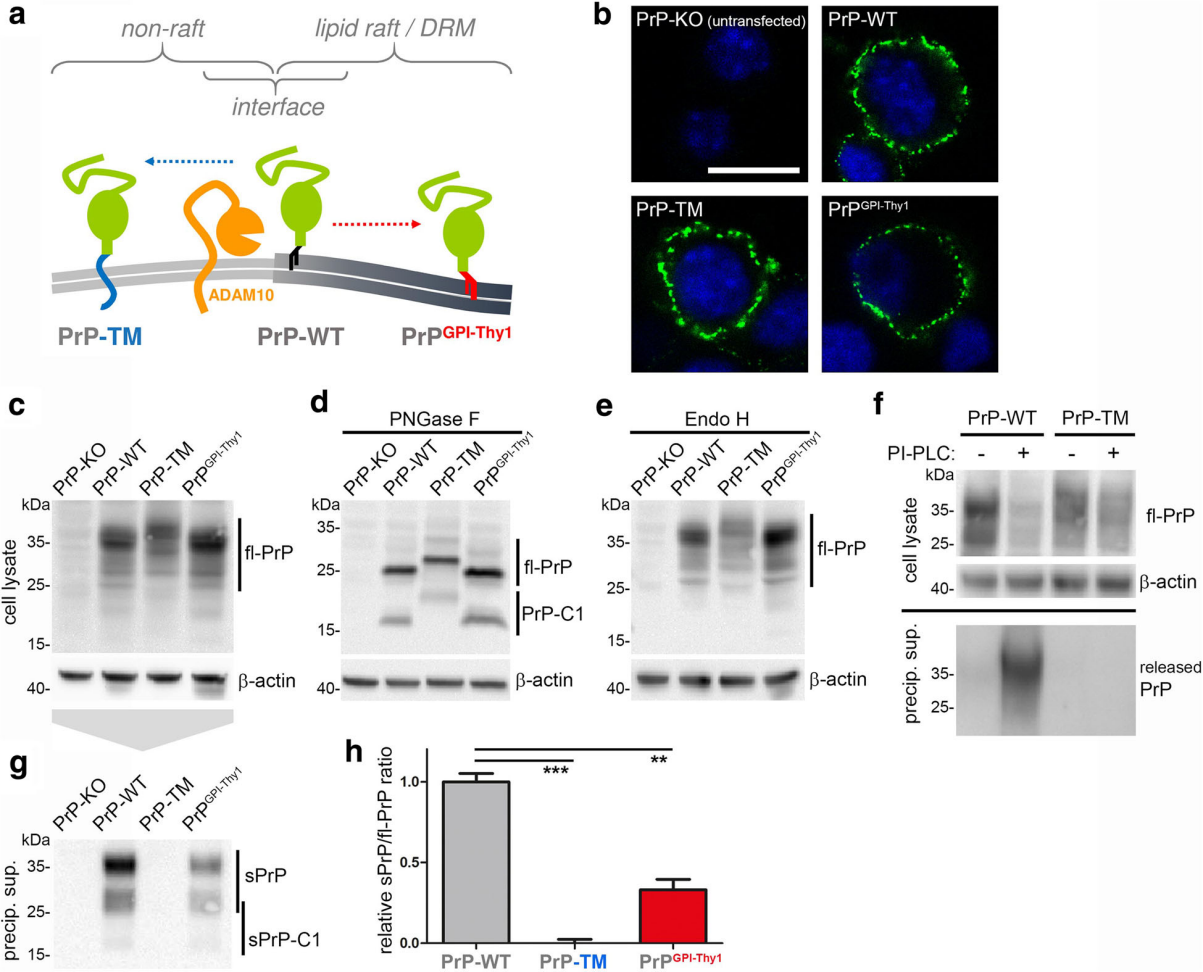
Membrane attachment and topology of PrP^C^ impact on its shedding. **a** Schematic representation of the expected subdomain localization of transmembrane PrP (PrP-TM; outside of lipid rafts) and PrP with an altered GPI-anchor signal sequence (PrP^GPI-Thy1^; in the core of lipid rafts) compared to wild-type PrP (PrP-WT; in the periphery of lipid rafts) at the plasma membrane. **b** Confocal microscopy showing that all PrP constructs are expressed and present at the cell surface upon transfection into PrP-KO N2a cells. **c**,**d**,**e** Representative western blots showing comparable expression of the constructs in lysates (**c**), revealing presence of both fl-PrP and C1 upon deglycosylation (**d**), and indicating correct processing and sorting as there is no altered glycopattern upon digestion of samples with Endo H (**e**). **f** Biochemical analysis upon incubation of cells with phospholipase C (PI-PLC) to cleave GPI-anchor structures. For PrP-WT, a reduction in cell-associated fl-PrP is accompanied by appearance of PrP in supernatants. Lack of this release in the case of PrP-TM confirms absence of a GPI-anchor and supports membrane attachment via a transmembrane domain. **g** Representative western blot analysis using the sPrP^G228^ Ab reveals lack of shed PrP for PrP-TM and reduced shedding in cells expressing PrP^GPI-Thy1^ compared to PrP-WT. Quantification of the ratio of shed PrP (**g**) referred to fl-PrP (**c**) is shown in H (PrP-WT was set to one; *p* = 0.00005 for PrP-TM to PrP-WT; *p* = 0.001 for PrP^GPI-Thy1^ to PrP-WT; n = 3)

An additional modification that influences membrane topology, biological functions and pathophysiological roles of the prion protein is the type of membrane attachment. With PrP^C^ being one of only few GPI-anchored substrates of ADAM10 [47, 87], we wondered how altered attachment and topology at the membrane would affect its shedding. To this end, we used mutants of PrP^C^ either comprising a transmembrane domain instead of the GPI-anchor (PrP-TM) or carrying the GPI-anchor signal sequence (and –as a likely consequence– the GPI-anchor [61] (Puig et al. submitted)) of Thy-1, a protein described to reside in the dense cores of detergent-resistant membranes (herein referred to as lipid rafts) [88]. Whereas interaction between ADAM10 and PrP^C^ is thought to occur at the interface between lipid rafts and non-raft regions (Fig. 4a), previous studies have shown that PrP-TM is relocated outside of rafts and turned signaling-incompetent [38, 63, 89] while PrP^GPIThy-1^ remains in rafts yet therein shows a different localization than PrP-WT [61] (Puig et al. submitted).

Immunofluorescent stainings of non-permeabilized cells showed surface expression (Fig. 4b) while western blot analysis revealed comparable expression of all constructs transfected into PrP-KO N2a cells (Fig. 4c). Deglycosylation of samples showed that all PrP mutants are subject to α-cleavage and confirmed an increase in molecular weight for PrP-TM due to its transmembrane domain (Fig. 4d). No alterations in the banding pattern were observed upon treatment with Endo H indicating correct glycosylation and –again– surface transport of all mutants (Fig. 4e). Lack of signal in the media for PrP-TM upon incubation of cells with PI-PLC proved the absence of a GPI-anchor and its attachment via a transmembrane domain (Fig. 4f).

Of note, despite a conserved shedding site in all constructs, shedding was completely abolished for PrP-TM (mean: 0 ± 0.02; *n* = 3; SEM) and significantly reduced for PrP^GPIThy-1^ (0.33 ± 0.06) compared to PrP-WT (set to 1.00 ± 0.05) (Fig. 4g,h). In conclusion, altered membrane attachment and, hence, changed membrane localization severely impact on PrP^C^ shedding.

### Shedding is part of a compensatory cellular network regulating PrP^C^ homeostasis

**Fig. 5:**
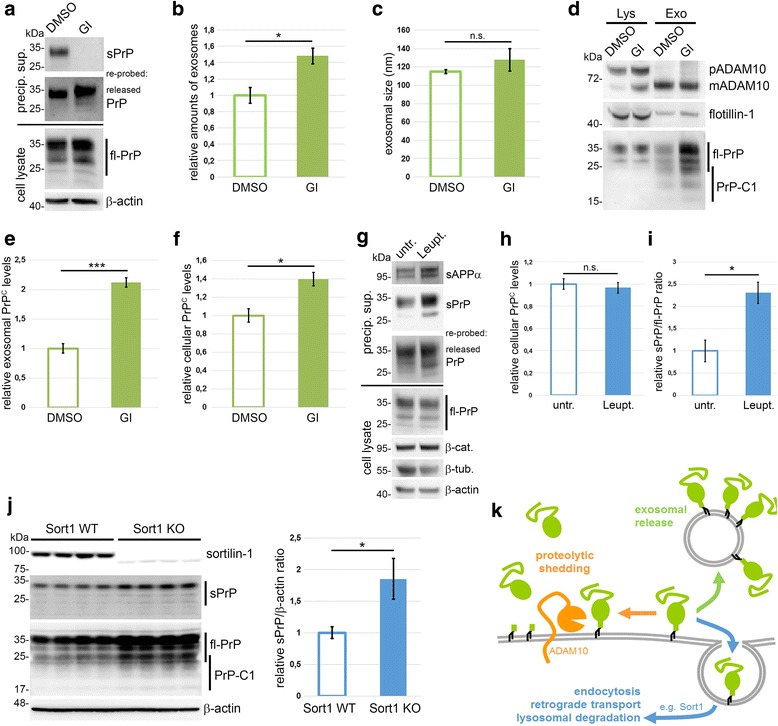
Shedding plays a key role in PrP^C^ homeostasis of a cell. **a** Representative western blot of N2a cells treated with the ADAM10 inhibitor GI254023X (GI; or DMSO as control). No sPrP is detected upon GI treatment yet re-probing the blot with POM2 Ab indicates release of PrP by other routes. **b** Quantification of relative amounts and (**c**) average size of exosomes released by N2a cells upon treatment with GI. Nanoparticle tracking analysis shows a significant increase in exosomal release (*n* = 4; *p* = 0.011) while the size of exosomes is not altered (n = 4). **d** After GI or DMSO treatment, cell lysates (Lys) and normalized amounts of exosomes (Exo) were analysed by western blot. Note that only mature ADAM10 is sorted into exosomes. Quantifications of relative exosomal PrP^C^ load (**e**; n = 3; *p* = 0.0006) and PrP^C^ levels in cell lysates (**f**; n = 3; *p* = 0.019) upon GI (or DMSO) treatment. PrP^C^ signals were referred to flotillin signal for quantification. **g** Representative western blot of N2a cells treated with lysosomal inhibitor Leupeptin (Leupt.) or left untreated (untr.) showing levels of sAPPα, sPrP and released PrP in media supernatants as well as cell-associated fl-PrP and cytosolic proteins β-catenin, β-tubulin and β-actin in lysates. Quantification shows no alterations of relative PrP levels in lysates (**h**; n = 3) yet a significant increase in PrP shedding (**i**; n = 3; *p* = 0.018) caused by lysosomal inhibition. **j** Western blot analysis of brain homogenates of sortillin-1-deficient (Sort1 KO) and control mice (Sort1 WT). Sort1 KO mice present with elevated levels of both sPrP and fl-PrP. Quantification of relative levels of sPrP (referred to actin) is shown (n = 4; *p* = 0.0103). **k** Schematic drawing summarizing three compensatory arms in the regulation of cellular PrP levels: ADAM10-mediated shedding (orange arrow), exosomal release (green arrow) and lysosomal targeting and degradation (blue arrow). Scheme makes no claim to completeness as other important factors regulating PrP^C^ levels, such as transcriptional/translational control, are not depicted here

As already shown in Fig. 1f, we consistently observed an increased cellular release of PrP via alternative routes whenever the proteolytic shedding was impaired (e.g. by inhibition of ADAM10 with GI; Fig. 5a). Since PrP^C^ is released via microvesicles (e.g. exosomes [72, 90, 91]), we wondered whether this mechanism could compensate for abolished shedding. Inhibition of ADAM10 with GI resulted in a significant rise of exosome release (mean: 1.48 ± 0.09; compared to controls set to 1.00 ± 0.09; *n* = 4; SEM; Fig. 5b) without affecting the typical size of these vesicles (mean: 128 ± 12 nm; compared to controls 115 ± 2 nm; n = 4; SEM; Fig. 5c; details for characterization of exosomes are shown in Additional file 7). When amounts of exosomes were normalized, we found more than a twofold increase in their average PrP^C^ load upon GI treatment (2.12 ± 0.08; compared to controls set to 1.00 ± 0.08; *n* = 3; SEM; Fig. 5d,e). PrP^C^ levels in the corresponding cells were only moderately, yet significantly increased (1.40 ± 0.07; compared to controls set to 1.00 ± 0.07; n = 3; SEM; Fig. 5d,f). Thus, as a consequence of impaired shedding, more PrP^C^ is packed into exosomes and more exosomes are released by N2a cells. Interestingly, such an alternative release of PrP^C^ was not observed in murine fibroblasts (MEF) lacking ADAM10 (Additional file 8) [71] which instead accumulated PrP^C^ to 3-fold the amount of wild-type MEFs (ADAM10 KO: 3.07 ± 0.12; WT set to 1.00 ± 0.12; *n* = 8; SEM). This may indicate that not all cell types possess the compensatory machinery ensuring PrP^C^ release and membrane homeostasis.

Finally, we investigated how degradation, as a third aspect involved in cellular PrP^C^ homeostasis, influences PrP^C^ shedding. We blocked lysosomal degradation by treatment of N2a cells with leupeptin. As expected, cytosolic proteins (e.g. β-actin, β-tubulin, β-catenin) known to be degraded by the proteasome rather than in lysosomes were not accumulated (Fig. 5g). Instead, increased secretion of sAPPα, the proteolytic fragment of APP, indicated successful lysosomal inhibition (Fig. 5g). Of note, cell-associated PrP^C^ levels remained rather stable despite this treatment (Leupt.: 0.97 ± 0.05; untr. Cells set to 1.00 ± 0.05; n = 3; SEM; Fig. 5g,h) yet shedding of PrP^C^ was significantly increased (Leupt.: 2.31 ± 0.24; untr. Cells set to 1.00 ± 0.24; n = 3; SEM; Fig. 5g,i). No obvious differences in alternatively released PrP (Fig. 5g) suggests that, in this condition, shedding is the main contributor avoiding increased cellular PrP^C^ levels.

Impaired lysosomal degradation and increased PrP^C^ levels have recently been shown in mice lacking the sorting receptor sortilin-1 [69]. As a consequence of hindered transport to lysosomes, these mice had shown increased PrP^Sc^ conversion and shortened survival when infected with prions. Given the increase in shedding upon lysosomal inhibition with leupeptin in cells shown before, we asked whether shedding of PrP^C^ is likewise affected by the impaired degradation due to lack of sortilin-dependent transport in vivo. In fact, we found an approximately 2-fold increase for sPrP in sortilin1-deficient mice compared to controls (Sort1 KO: 1.85 ± 0.32; Sort1 WT set to 1.00 ± 0.09; *n* = 4; SD; Fig. 5j). Rather than from up-regulation of sheddase activity, this increase seems to result from elevated cellular PrP^C^ levels caused by impaired degradation (Additional file 9). Nevertheless, this demonstrates the capability of ADAM10 to release increased amounts of substrate.

In summary, these data suggest ADAM10-mediated shedding as a relevant factor regulating PrP^C^ levels. Shedding, exosomal release and degradation of PrP^C^ may be interconnected mechanisms that act in a compensatory manner ensuring PrP^C^ homeostasis and allowing –if at all– only subtle changes thereof.

## Discussion

Evolutionary conserved proteolytic processing of the prion protein has been described a quarter of a century ago [92–94] (reviewed in [28]). However, we are just beginning to appreciate the physiological and pathological relevance of such cleavage events, which is partially due to technical difficulties in reliable detection of the respective fragments. We here present a novel antibody that detects shed PrP with high specificity and sensitivity in different applications. Despite the existence of several valuable antibodies against various epitopes in PrP^C^ (e.g. the POM antibodies used in this study [73]), until now it has only been possible to detect shed PrP upon rather labor-intensive and error-prone immunoprecipitation from or strong concentration of cell culture supernatants [47, 48]. And even that way, contribution of PrP^C^ released from cells by other routes (e.g. via exosomes) [91] to respective signals has to be considered. In tissue samples it has so far been impossible to specifically detect shed PrP due to the excess amounts of cell- or extracellular vesicle-associated fl-PrP^C^ of similar molecular weight masking any signal coming from the fraction of proteolytically shed PrP. This resulted in a lack of in vivo insight. These problems have been overcome and novel findings have been made with the new antibody.

Despite confirming antibody specificity, the absence of shed PrP in forebrain homogenates from mice with a depletion of ADAM10 in forebrain neurons to our surprise indicates that, at least under physiological conditions, no other cell types in the brain contribute to shedding in a detectable manner. It also questions a shedding of (neuronal) PrP^C^ *in trans* (e.g. by adjacent glia cells not depleted of the protease), a mechanism that has been shown for the ADAM10 substrate ephrin in HEK cells [95]. Our findings of abolished shedding in the absence of ADAM10 or upon pharmacological inhibition of ADAM10 also indicate that no other protease compensates for these manipulations in vitro or in vivo. Further support comes from mice coexpressing dominant negative ADAM10 with endogenous ADAM10, where we found a comparably strong (~ 50%) reduction in PrP shedding. Instead, previous western blot analyses of sAPPα in ADAM10 d.n. mice only showed a reduction of ~ 25% [96, 97] hinting at the known contribution of ADAM17/TACE in the cleavage of APP [98]. It should be considered that cleavage by another protease at a slightly different cleavage site would prevent detection with our antibody. However, our previous results obtained by pull-down of shed PrP from media of primary ADAM10 knockout neurons with classical PrP antibodies [48], together with a recent biophysical study [31], and the lack of any other reported candidate protease linked to the membrane-proximate shedding of PrP^C^, support the view of ADAM10 as the only relevant sheddase of PrP^C^. This is in clear contrast to the cleavage of other typical ADAM10 substrates such as APP, which –as mentioned above– to varying degrees and dependent on the experimental paradigm, can also be processed by other proteases [71, 99–101].

Our analysis suggests that diglycosylated PrP^C^ is the preferred glycoform to be shed by ADAM10, whereas mono- and especially unglycosylated forms seem to be relatively disfavored. Our data also indicates that this finding not simply results from differences in the availability of individual glycoforms as substrates at the plasma membrane under normal conditions (our transfected glycomutants as well as PrP^C^ in cells treated with TM or SWA were by all means localized at the surface). An alternative explanation could be that shed diglycosylated PrP is more protected than the other shed forms from potential cellular uptake and degradation and, thus, more abundant. In any case, among all soluble PrP^C^ fragments released from the cell by the proteolytic cleavages described to date, shed PrP is the only one that is glycosylated. As discussed earlier [28] this may well impact its binding affinities to both, toxic extracellular oligomers as well as physiological binding partners (e.g. surface signaling receptors), and thus define its specific biological functions. Moreover, by the predominantly diglycosylated state, physiologically shed PrP clearly differs from anchorless, mainly unglycosylated PrP of transgenic mice used in several seminal prion inoculation studies in the past [54, 102–104]. This difference has to be considered and, in the context of prion diseases, might explain why transgenic anchorless PrP efficiently converts to PrP^Sc^ and can even spontaneously form prions [54, 103], whereas shed PrP rather seems to block PrP^Sc^ formation in mice [50]. Fittingly, the N-glycans are known to influence transmissibility and conversion to PrP^Sc^ [62, 80–83].

Altered shedding efficiency for different glycoforms, however, might in part also be caused by a different sorting given that the glycans have a significant impact on the polarized trafficking of PrP^C^ in MDCK cells [61]. Despite a role for the N-glycans, we also demonstrated that changes in the type of membrane anchorage and, as a likely consequence, altered membrane topology affects shedding. Shifting PrP^C^ outside of rafts by addition of a transmembrane domain [63, 89] completely abolished the shedding while the assumed re-localization of PrP^C^ within lipid rafts via exchange of the GPI-anchor signal sequence [61, 88] reduced shedding to ~ 30% in N2a cells. The latter is in good agreement with unpublished data obtained in transgenic mice expressing the same PrP^GPIThy-1^ construct (Puig et al., submitted). Instead of changing the anchorage of the substrate as done here, others have changed membrane attachment of the protease [105]. Lipid raft targeting of ADAM10 by addition of a GPI-anchor in that study severely affected APP processing. Unfortunately, processing of PrP^C^ was not investigated there.

Since ADAM10 is mainly located outside of lipid rafts [105, 106], whereas PrP^C^ is a resident of these microdomains, transient interaction between protease and substrate (presumably regulated by accessory proteins such as tetraspanins [107–109]) and cleavage is thought to occur at the periphery of rafts. This molecular get-together might further be supported by the capacity of PrP^C^ to leave and re-enter lipid rafts in a constitutive manner [85, 110, 111]. Our findings also suggest an impact of the flexible N-terminal part of PrP^C^ on the shedding efficiency. Whether this unanticipated influence is due to sterical aspects or rather reflects the role of regulatory binding partners known to especially interact with the N-terminal half of PrP^C^ [5, 27, 112], deserves further investigations.

Our data indicate that shedding is a relevant mechanism embedded in a compensatory machinery ensuring homeostasis of PrP^C^. In neurons and neuronal cells, this system (involving proteolytic and exosomal release as well as trafficking to lysosomes) seems to ensure that cell-associated PrP^C^ levels are kept stable or –at most– increase twofold upon perturbation (as indicated in some experiments of this study and observed in neurons or mice lacking the sheddase ADAM10 [48, 50] or the transport factor sortilin-1 [69]). Interestingly, a recent study showed that exosomal release is controlled by PrP^C^ membrane levels [113]. Though clearly requiring further investigation, it might be speculated that other cell types, such as fibroblasts studied here, do not possess the system to compensate for such perturbation in one of the mechanisms discussed above, and consequently accumulate PrP^C^ to higher levels.

Manipulation of PrP^C^ shedding is feasible and might be of therapeutic interest. Despite the challenge by possible side effects due to the broad spectrum of ADAM10 substrates, one obvious question then is into which direction to modify PrP^C^ shedding [87, 114].

With regard to neurodegenerative proteinopathies, such as Alzheimer’s or prion diseases, stimulation of this cleavage will likely be beneficial. First, it reduces PrP^C^ levels at the cell surface and may thereby lower neurotoxicity. Moreover, several studies showed that soluble PrP targets toxic oligomers and fibrils in the extracellular space [55–58, 115]. In prion diseases, shedding efficiency inversely correlates with PrP^Sc^ formation [50, 52]. Notably, resveratrol, the drug that was used here to stimulate shedding, reduced PrP^Sc^ formation and prion infectivity in a recent study [116]. Whether this anti-prion efficacy is indeed related to shedding, remains to be investigated. Besides proteinopathies, positive effects of stimulated shedding can also be expected given the potential role of this fragment in neurite outgrowth [13, 14] and neuroprotection [15, 49]. In that way, the role of shed PrP is reminiscent of sAPPα, the APP-derived fragment also generated by ADAM10 [117].

Other pathological conditions, in contrast, may rather require inhibition of PrP^C^ shedding: it is intriguing that both, ADAM10 [118, 119] and PrP^C^, have been linked with immune signaling and chronic inflammatory processes [120, 121] as well as with tumorigenesis and cancer progression [122–124], where expression levels of these two proteins generally correlate with poor prognosis. Though this could well be unrelated co-incidence, it might also be speculated that these pathophysiological roles are partially related to the production of shed PrP. Of note, it is precisely shed PrP that was causally linked with chronic inflammatory neuropathology in HIV patients [60] and development of tumours in the central nervous system [59] in two recent studies. This further supports the relevance of shed PrP in different pathophysiological conditions and highlights the need for further studies on the ADAM10-mediated shedding of PrP^C^.

## Conclusion

Proteolytic shedding of the prion protein has most recently attracted scientific interest with regard to diverse pathological conditions affecting the brain. Using a novel antibody for the specific detection of shed PrP, we demonstrated structural and regulatory aspects influencing this cleavage and show that it can - in principle - be pharmacologically manipulated. The latter, together with the rather ubiquitous expression of PrP^C^ in several tissues and cell lines, as well as the lack of compensation by other proteases discussed above, also turns (i) PrP^C^ into an ideal “control” substrate, (ii) assessment of PrP^C^ shedding into a reliable “read out”, and (iii) our antibody into a valuable tool for any future studies investigating ADAM10-mediated cleavages and their pharmacological accessibility. With direct regard to the shedding of PrP^C^, both, therapeutic stimulation as well as inhibition, may be conceivable depending on the pathological context.

## Additional files

Additional file 1: (.jpg) The 3F4-tag in PrP^C^ does not alter the ADAM10-mediated shedding. Western blot analysis of forebrain homogenates comparing PrP^C^ shedding between mice expressing endogenous wild-type PrP^C^ (PrP^C^WT) and knock-in mice expressing 3F4-tagged PrP^C^ instead (PrP^3F4^KI). Quantification was done by referring the sPrP signal to the respective fl-PrP signal (POM2 Ab) of the re-probed blot and is shown on the right (*n* = 3). As in other parts of this study, forebrain homogenates of *Prnp*^0/0^, ADAM10 cKO and *tg*a20 mice served as specificity controls. The position of air bubbles on the membrane (indicated by arrows) further supports the slight molecular weight shift between sPrP and fl-PrP described in Fig. 1b. To prove genotypes of PrP^C^WT and PrP^3F4^KI mice, in a parallel blot shown below, PrP^C^ was first detected with an antibody directed against the 3F4 epitope and re-probed with POM2. (JPEG 225 kb)

Additional file 2: (.jpg) Species specificity of the sPrP^G228^ antibody. (A) Comparison of the C-terminal amino acid sequence of PrP^C^ in mouse (*Mus musculus*), rat (*Rattus norvegicus*) and rabbit (*Oryctolagus cuniculus*) (source: www.uniprot.org). “*NH*_*2*_-…-” indicates the N-terminal direction, “*-GPI*” the C-terminal GPI-anchor attachment site. Asterisks indicate position of ADAM10-mediated shedding in mice and rats with Gly228 representing the new C-terminus of shed PrP. Note the sequence difference compared with rabbit PrP^C^. (B) Western blot analysis of forebrain homogenates from different murine models (*tg*a20, *Prnp*^0/0^, wild-type (C57BL/6)) as well as from rat and rabbit. As expected for its epitope, the sPrP^G228^ antibody detects sPrP in mouse (*tg*a20 and wild-type) and rat, whereas the brain sample of rabbit only presents an immunoglobulin light chain (rb Ig-LC) signal at 25 kDa resulting from the anti-rabbit secondary antibody used for detection. Re-probing the blot with POM2 antibody reveals expression levels of PrP^C^. (JPEG 388 kb)

Additional file 3: (.jpg) Use of the sPrP^G228^ antibody for immunohistochemical stainings**.** Sagittal brain sections of a *Prnp*^0/0^, a wild-type (C57BL/6) and a *tg*a20 mouse stained with hematoxilin/eosin (H&E), an antibody against total PrP^C^ (SAF84), or the sPrP Ab showing the hippocampus (Hc) and parts of cortical areas (Cx) in overviews. Magnifications are shown for the corpus callosum and CA1 region (upper insets) as well as for the dentate gyros (DG) and CA3 region (lower insets) of the hippocampus. With the sPrP^G228^ Ab, a diffuse brownish staining of the brain parenchyma is seen for wild-type and *tg*a20 whereas *Prnp*^0/0^ brain only shows blue counterstaining. Comparison with the SAF84 staining reveals that levels of shed PrP correlate with overall PrP^C^ expression. Scale bars represent 100 μm. (JPEG 1948 kb)

Additional file 4: (.jpg) Differences in the glycopattern between total and cell surface PrP^C^ in N2a cells. (A) Western blot analysis and (B) densitometric quantification of glycoform proportions of total (cell lysates) versus cell surface fl-PrP (biotinylated samples) using POM2 antibody for detection. Absence of actin and almost exclusive expression of mature ADAM10 (with almost no premature ADAM10) in the biotinylated samples confirm technical soundness of the assay (the upwards shift of ADAM10 in gel likely results from the assay protocol). Quantification reveals that the fraction of diglycosylated PrP at the cell surface is increased compared to total PrP in cell lysates (diglycosylated: 68.0 ± 0.7% (surface PrP) vs. 55.1 ± 2.4% (total PrP); monoglycosylated: 23.5 ± 0.8% (surface PrP) vs. 29.3 ± 0.6% (total PrP); unglycosylated: 8.5 ± 0.2% (surface PrP) vs. 15.5 ± 1.9% (total PrP); n = 3; ±SD). (JPEG 610 kb)

Additional file 5: (.jpg) Preference for the shedding of fl-PrP over truncated C1 fragment indicates a role of the N-terminal part of PrP^C^. Western blot analysis of PrP-KO N2a cells transfected with PrP-WT or N-terminally truncated PrP-C1 (corresponding to physiological C1 fragment). Analysis of cell lysates (on the left) reveals N-glycosylation and comparable expression levels for both constructs. Actin served as loading control. Enzymatic deglycosylation (PNGase F) was performed and samples run on a parallel blot to confirm identity of constructs. POM1 antibody was used for detection of PrP^C^ in lysates. Corresponding cell culture supernatants were precipitated and run on a parallel blot (on the right) and shed PrP forms were detected with sPrP^G228^ antibody. Released sAPPα was detected as loading control for supernatants. Signal intensities of shed PrP forms (sPrP^G228^ Ab) were referred to total PrP signal intensities in lysates (POM1) and quantification reveals a significantly reduced shedding for PrP-C1 (relative ratio shed/total PrP: 0.12 ± 0.03) compared to (full-length) PrP-WT (set to 1.00 ± 0.11; *p* = 0.0017; n = 3; ±SEM). (JPEG 799 kb)

Additional file 6: (.jpg) Side-by-side comparison of SWA and TM treatments and enzymatic deglycosylation reactions. Western blot of untreated (untr.), SWA- or TM-treated N2a cells showing lysates without (−) or with (+) enzymatic treatment for differential deglycosylation (Endo H or PNGase F). As also shown in Fig. 3a and b, TM-treatment causes a complete inhibition of PrP glycosylation, whereas SWA-treatment results in a shift in the banding pattern (compared to untreated cells) and (at least partial) Endo H sensitivity due to inhibition of complex glycosylation. Changes in the glycopattern and running behaviour support the functioning of our enzymatic deglycosylation protocols also performed for the experiments shown in Figs. 2, 3 and 4. Actin is shown as a loading control. (JPEG 221 kb)

Additional file 7: (.jpg) Exosome characterization using the NanoSight system. Representative experiment showing the raw data of 10 serial measures (upper curves) and the averaged data (lower curves) derived from media supernatants of DMSO- or GI254023X (GI)-treated N2a cells. X-axis: size (nm); Y-axis: concentration (E6 particles/ml). Blue numbers at the tips of curves represent mean sizes. (JPEG 348 kb)

Additional file 8: (.jpg) Embryonic fibroblasts (MEF) of ADAM10 knockout mice accumulate PrP^C^. Representative western blot of media supernatants and lysates of wild-type (WT) and ADAM10 KO MEF. Lack of shedding and no increased compensatory release of PrP is observed in ADAM10 KO cells. ADAM10 is shown in lysates as a proof of genotypes. Increased levels of PrP^C^ are found in lysates and quantified by referring to β-actin (*n* = 8; *p* = 0.00005). (JPEG 131 kb)

Additional file 9: (.jpg) Quantification of fl-PrP levels and ratio of sPrP/fl-PrP in Sort1 knockout mice. These quantifications refer to main Fig. 5j. (A) Increased amounts of fl-PrP are found in brains of Sort1 KO mice (2.11 ± 0.23; *p* = 0.0004; *n* = 4) compared to controls (WT set to 1.00 ± 0.21; SD). Actin served as loading control and for reference in densitometric quantification. (B) No significant differences are detected in the ratio of sPrP to fl-PrP between Sort1 KO mouse brains (0.85 ± 0.07, *p* = 0.128; n = 4) and controls (WT set to 1.00 ± 0.14). (JPEG 229 kb)

## Abbreviations

Ab: antibody
ADAM: a disintegrin and metalloproteinase
AIDS: acquired immunodeficiency syndrome
APP: Amyloid precursor protein
Aβ: Amyloid-beta peptide
BSA: bovine serum albumin
BSE: bovine spongiform encephalopathy
cKO: conditional knockout
d.n.: dominant negative
DMSO: dimethyl sulfoxide
FBS: fetal bovine serum
fl-PrP: full length prion protein
GI: GI254023X (ADAM10 inhibitor)
GPI: glycosylphosphatidylinositol
HIV: human immunodeficiency virus
KI: knock-in
KO: knockout
MDCK: Madin-Darby Canine Kidney (cells)
MEF: murine embryonal fibroblasts
NaDOC: sodium deoxycholate
PBS: phosphate buffered saline
PI: protease inhibitor
PI-PLC: phosphatidylinositol-specific phospholipase C
*Prnp*^0/0^: PrP^C^ knockout
PrP^C^: cellular prion protein
PrP^Sc^: pathological (Scrapie) isoform of the prion protein
PrP-TM: transmembrane PrP
Sort1: sortilin-1
sPrP: shed PrP
sPrP^G228^: antibody against shed PrP
SWA: swainsonine
TACE: tumor necrosis factor α converting enzyme (ADAM17)
TALEN: Transcription Activator-like Effector Nuclease
TBS: Tris-buffered saline
TCA: trichloroacetic acid
*tg*a20: prion protein overexpressing mouse line
TM: Tunicamycin
WT: wild-type
