# Supplementary figures and images for: Structural and mechanistic aspects influencing the ADAM10-mediated shedding of the prion protein
Source: Mol Neurodegener. 2018 Apr 6;13:18. doi: 10.1186/s13024-018-0248-6 (PMC5889536; doi:10.1186/s13024-018-0248-6)

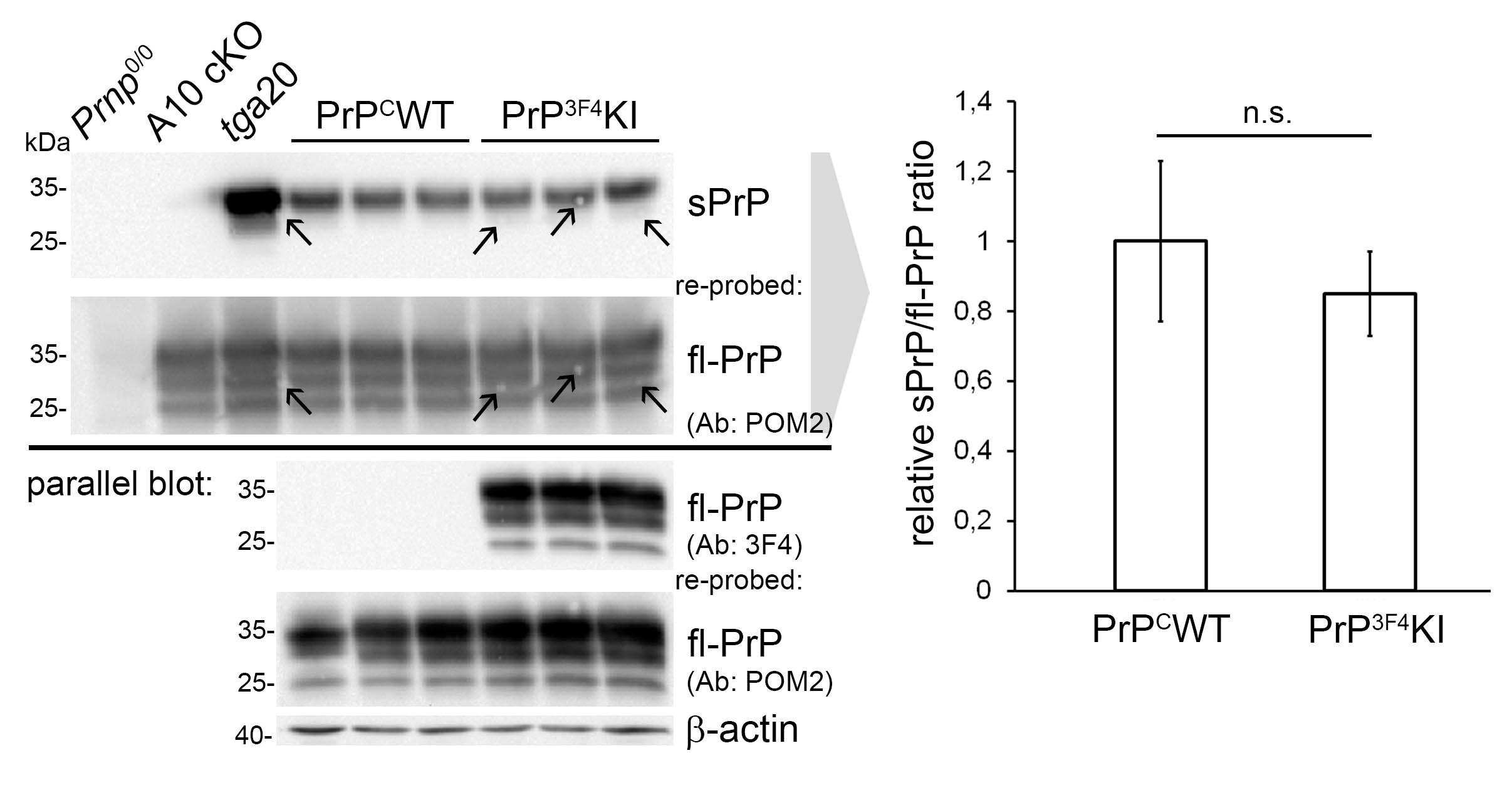

Supplement: Supplementary file 1 — (.jpg) The 3F4-tag in PrPC does not alter the ADAM10-mediated shedding. Western blot analysis of forebrain homogenates comparing PrPC shedding between mice expressing endogenous wild-type PrPC (PrPCWT) and knock-in mice expressing 3F4-tagged PrPC instead (PrP3F4KI). Quantification was done by referring the sPrP signal to the respective fl-PrP signal (POM2 Ab) of the re-probed blot and is shown on the right (n = 3). As in other parts of this study, forebrain homogenates of Prnp0/0, ADAM10 cKO and tga20 mice served as specificity controls. The position of air bubbles on the membrane (indicated by arrows) further supports the slight molecular weight shift between sPrP and fl-PrP described in Fig. 1b. To prove genotypes of PrPCWT and PrP3F4KI mice, in a parallel blot shown below, PrPC was first detected with an antibody directed against the 3F4 epitope and re-probed with POM2. (JPEG 225 kb) [file 13024_2018_248_MOESM1_ESM.jpg]

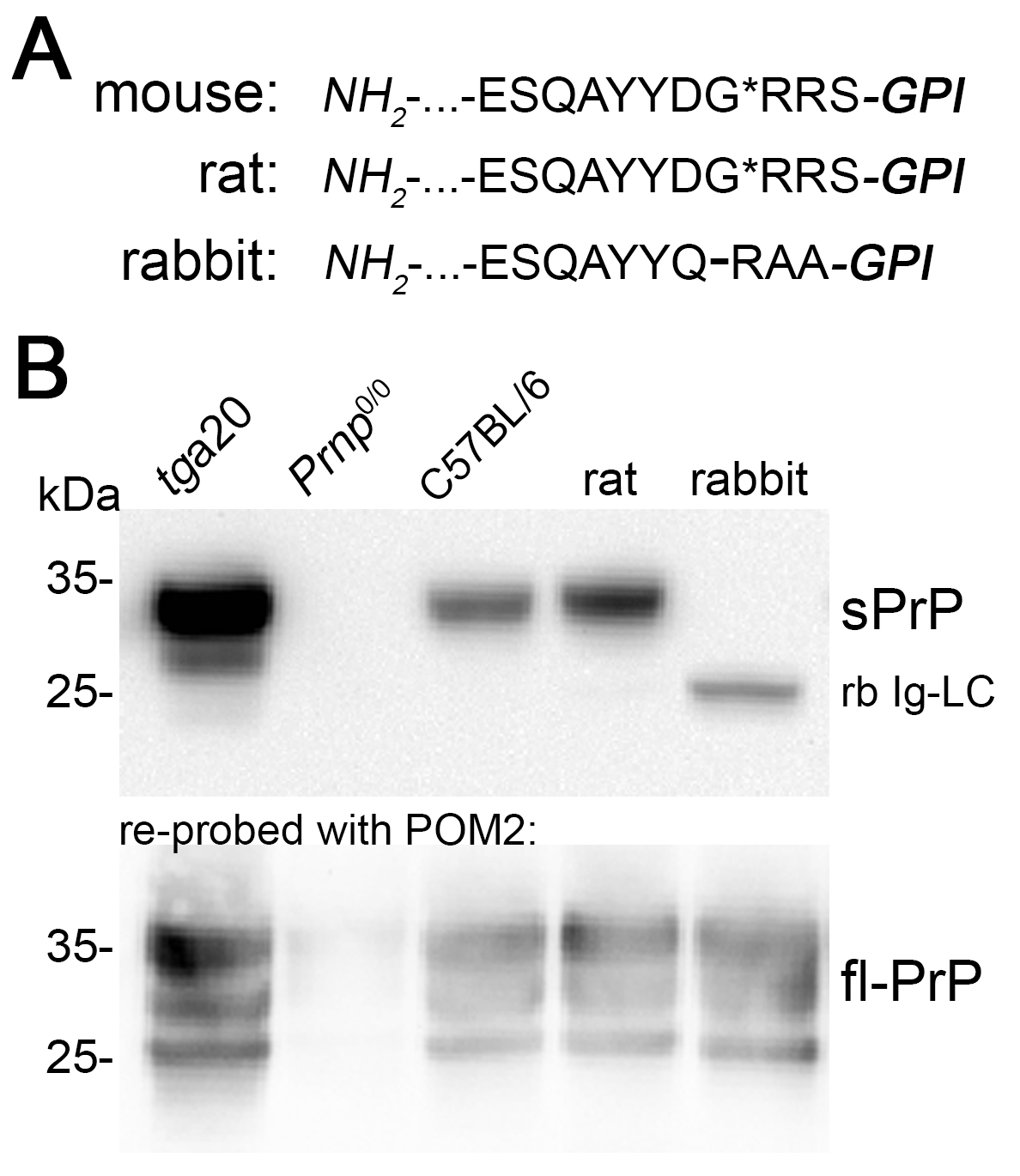

Supplement: Supplementary file 2 — (.jpg) Species specificity of the sPrPG228 antibody. (A) Comparison of the C-terminal amino acid sequence of PrPC in mouse (Mus musculus), rat (Rattus norvegicus) and rabbit (Oryctolagus cuniculus) (source: www.uniprot.org). “NH2-…-” indicates the N-terminal direction, “-GPI” the C-terminal GPI-anchor attachment site. Asterisks indicate position of ADAM10-mediated shedding in mice and rats with Gly228 representing the new C-terminus of shed PrP. Note the sequence difference compared with rabbit PrPC. (B) Western blot analysis of forebrain homogenates from different murine models (tga20, Prnp0/0, wild-type (C57BL/6)) as well as from rat and rabbit. As expected for its epitope, the sPrPG228 antibody detects sPrP in mouse (tga20 and wild-type) and rat, whereas the brain sample of rabbit only presents an immunoglobulin light chain (rb Ig-LC) signal at 25 kDa resulting from the anti-rabbit secondary antibody used for detection. Re-probing the blot with POM2 antibody reveals expression levels of PrPC. (JPEG 388 kb) [file 13024_2018_248_MOESM2_ESM.jpg]

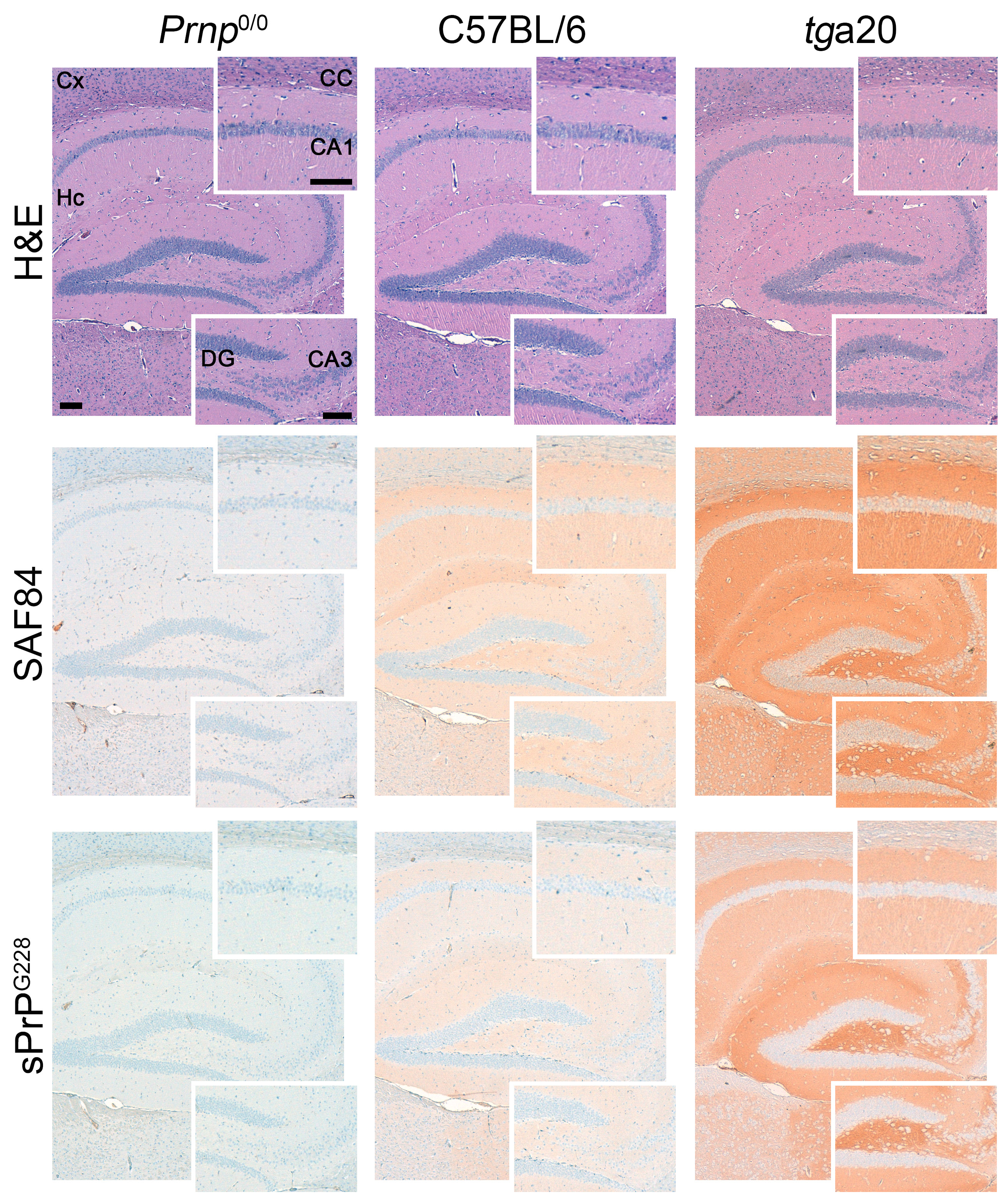

Supplement: Supplementary file 3 — (.jpg) Use of the sPrPG228 antibody for immunohistochemical stainings. Sagittal brain sections of a Prnp0/0, a wild-type (C57BL/6) and a tga20 mouse stained with hematoxilin/eosin (H&E), an antibody against total PrPC (SAF84), or the sPrP Ab showing the hippocampus (Hc) and parts of cortical areas (Cx) in overviews. Magnifications are shown for the corpus callosum and CA1 region (upper insets) as well as for the dentate gyros (DG) and CA3 region (lower insets) of the hippocampus. With the sPrPG228 Ab, a diffuse brownish staining of the brain parenchyma is seen for wild-type and tga20 whereas Prnp0/0 brain only shows blue counterstaining. Comparison with the SAF84 staining reveals that levels of shed PrP correlate with overall PrPC expression. Scale bars represent 100 μm. (JPEG 1948 kb) [file 13024_2018_248_MOESM3_ESM.jpg]

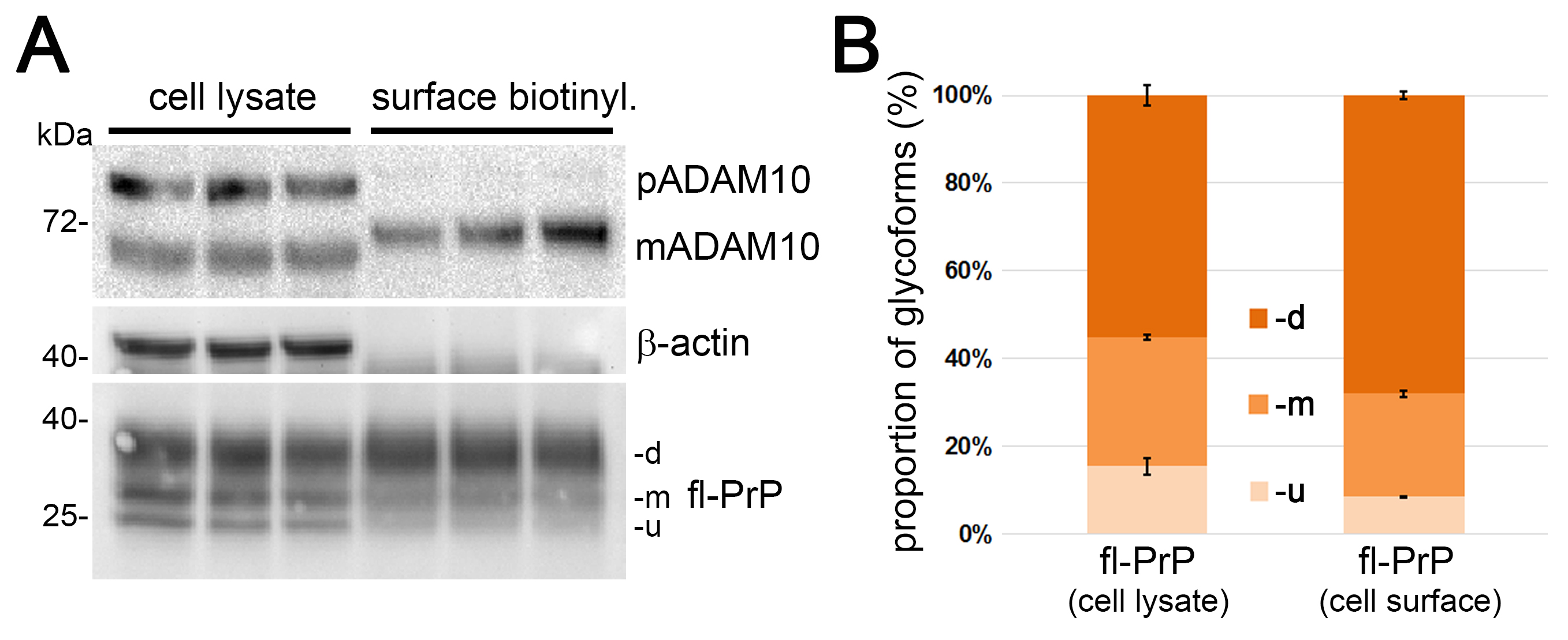

Supplement: Supplementary file 4 — (.jpg) Differences in the glycopattern between total and cell surface PrPC in N2a cells. (A) Western blot analysis and (B) densitometric quantification of glycoform proportions of total (cell lysates) versus cell surface fl-PrP (biotinylated samples) using POM2 antibody for detection. Absence of actin and almost exclusive expression of mature ADAM10 (with almost no premature ADAM10) in the biotinylated samples confirm technical soundness of the assay (the upwards shift of ADAM10 in gel likely results from the assay protocol). Quantification reveals that the fraction of diglycosylated PrP at the cell surface is increased compared to total PrP in cell lysates (diglycosylated: 68.0 ± 0.7% (surface PrP) vs. 55.1 ± 2.4% (total PrP); monoglycosylated: 23.5 ± 0.8% (surface PrP) vs. 29.3 ± 0.6% (total PrP); unglycosylated: 8.5 ± 0.2% (surface PrP) vs. 15.5 ± 1.9% (total PrP); n = 3; ±SD). (JPEG 610 kb) [file 13024_2018_248_MOESM4_ESM.jpg]

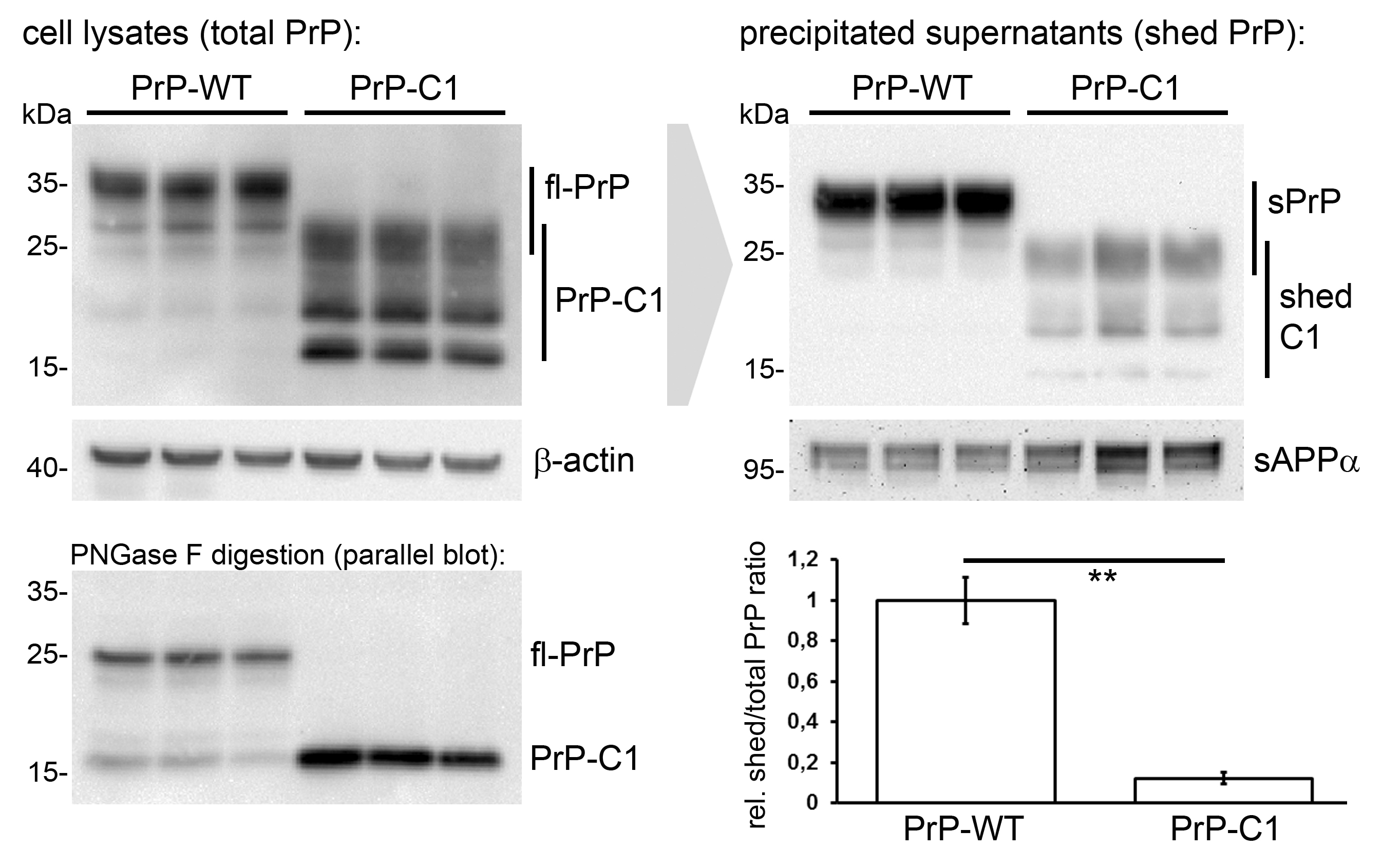

Supplement: Supplementary file 5 — (.jpg) Preference for the shedding of fl-PrP over truncated C1 fragment indicates a role of the N-terminal part of PrPC. Western blot analysis of PrP-KO N2a cells transfected with PrP-WT or N-terminally truncated PrP-C1 (corresponding to physiological C1 fragment). Analysis of cell lysates (on the left) reveals N-glycosylation and comparable expression levels for both constructs. Actin served as loading control. Enzymatic deglycosylation (PNGase F) was performed and samples run on a parallel blot to confirm identity of constructs. POM1 antibody was used for detection of PrPC in lysates. Corresponding cell culture supernatants were precipitated and run on a parallel blot (on the right) and shed PrP forms were detected with sPrPG228 antibody. Released sAPPα was detected as loading control for supernatants. Signal intensities of shed PrP forms (sPrPG228 Ab) were referred to total PrP signal intensities in lysates (POM1) and quantification reveals a significantly reduced shedding for PrP-C1 (relative ratio shed/total PrP: 0.12 ± 0.03) compared to (full-length) PrP-WT (set to 1.00 ± 0.11; p = 0.0017; n = 3; ±SEM). (JPEG 799 kb) [file 13024_2018_248_MOESM5_ESM.jpg]

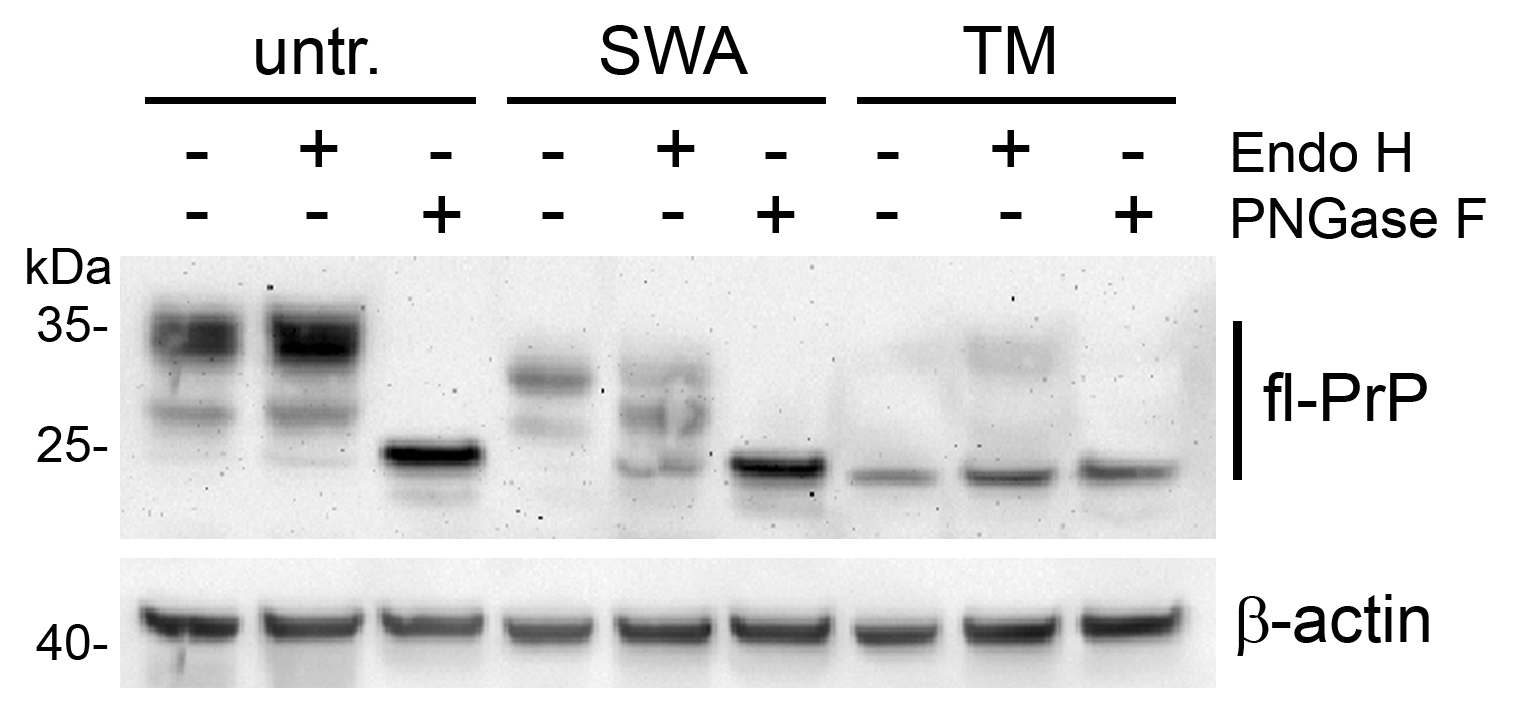

Supplement: Supplementary file 6 — (.jpg) Side-by-side comparison of SWA and TM treatments and enzymatic deglycosylation reactions. Western blot of untreated (untr.), SWA- or TM-treated N2a cells showing lysates without (−) or with (+) enzymatic treatment for differential deglycosylation (Endo H or PNGase F). As also shown in Fig. 3a and b, TM-treatment causes a complete inhibition of PrP glycosylation, whereas SWA-treatment results in a shift in the banding pattern (compared to untreated cells) and (at least partial) Endo H sensitivity due to inhibition of complex glycosylation. Changes in the glycopattern and running behaviour support the functioning of our enzymatic deglycosylation protocols also performed for the experiments shown in Figs. 2, 3 and 4. Actin is shown as a loading control. (JPEG 221 kb) [file 13024_2018_248_MOESM6_ESM.jpg]

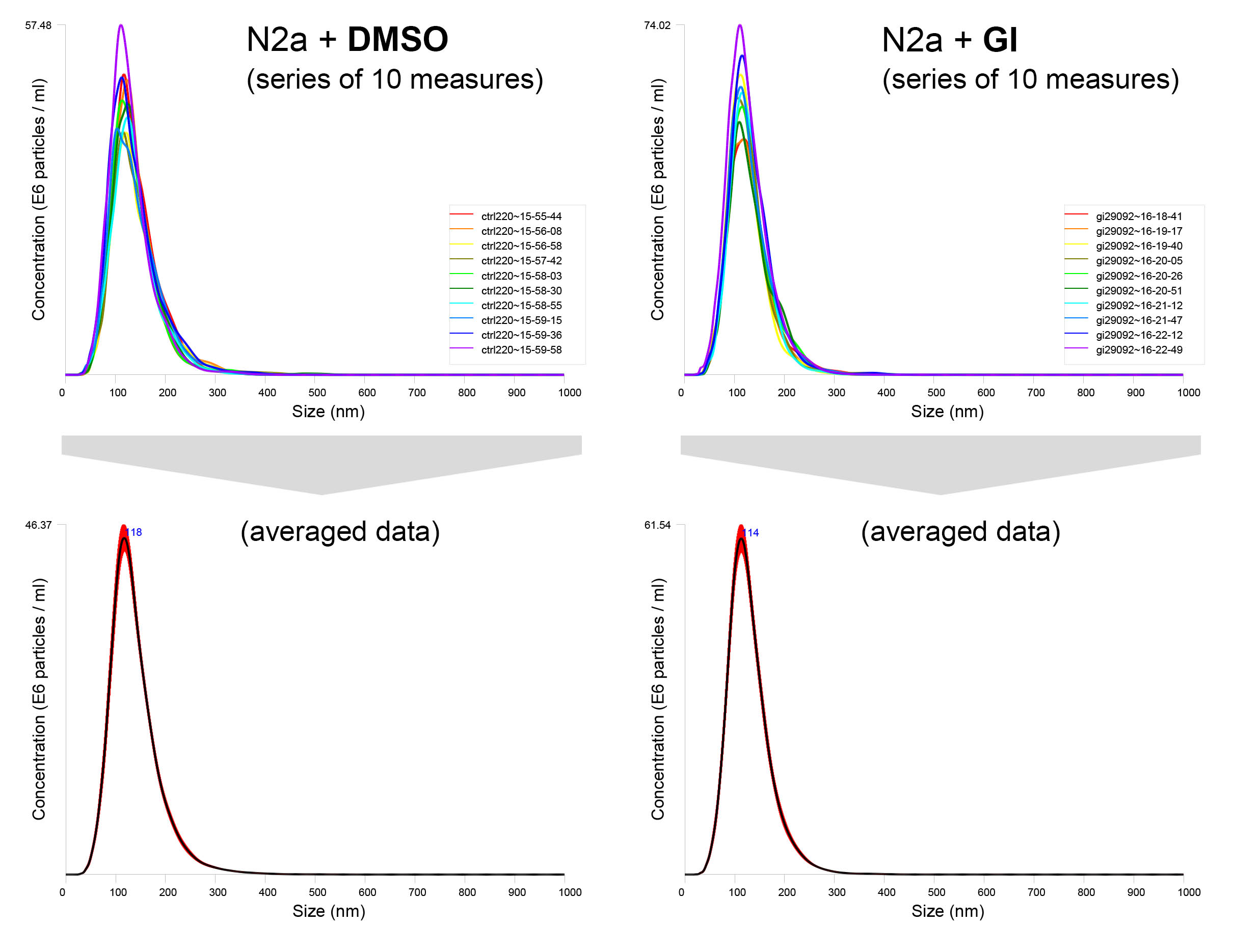

Supplement: Supplementary file 7 — (.jpg) Exosome characterization using the NanoSight system. Representative experiment showing the raw data of 10 serial measures (upper curves) and the averaged data (lower curves) derived from media supernatants of DMSO- or GI254023X (GI)-treated N2a cells. X-axis: size (nm); Y-axis: concentration (E6 particles/ml). Blue numbers at the tips of curves represent mean sizes. (JPEG 348 kb) [file 13024_2018_248_MOESM7_ESM.jpg]

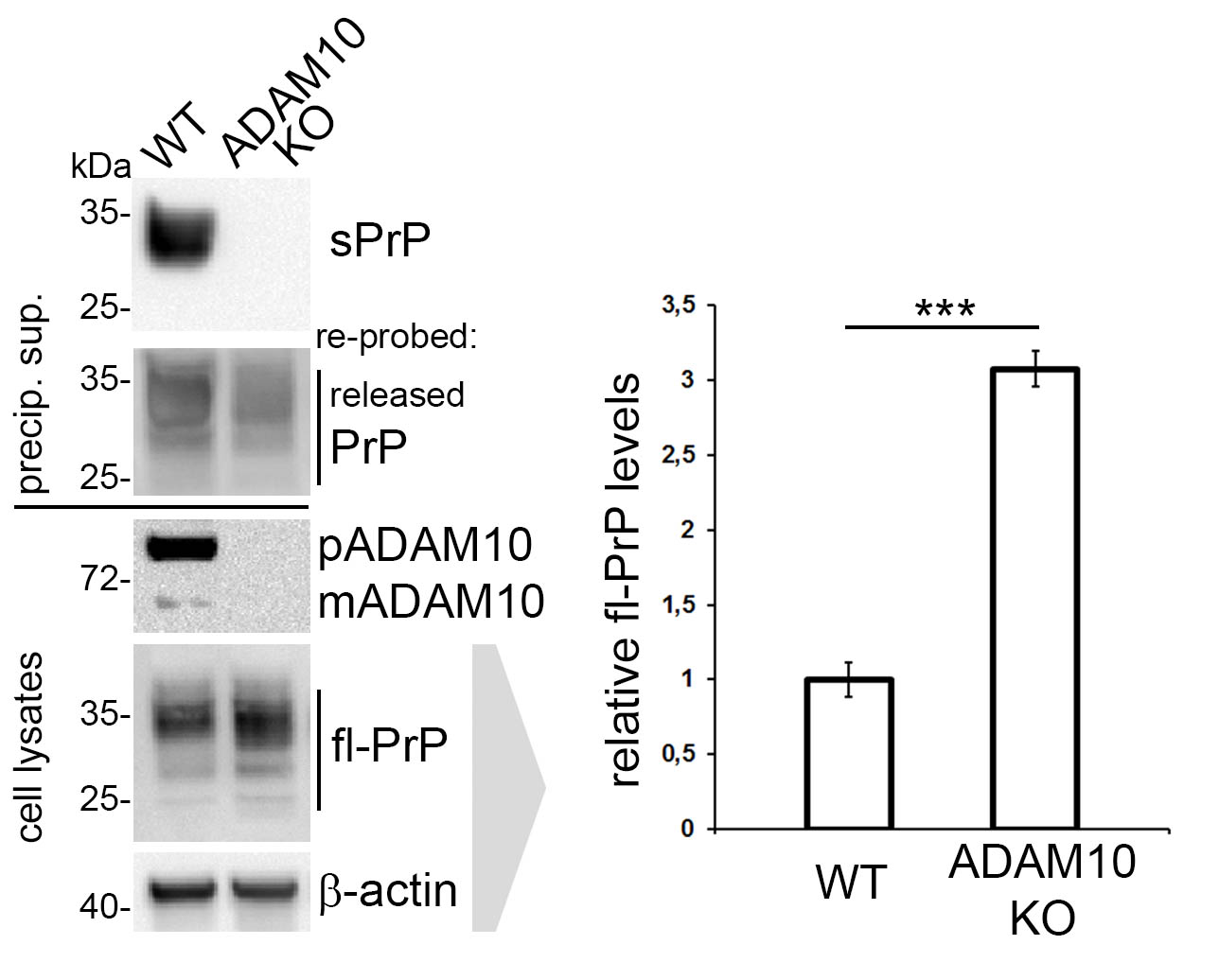

Supplement: Supplementary file 8 — (.jpg) Embryonic fibroblasts (MEF) of ADAM10 knockout mice accumulate PrPC. Representative western blot of media supernatants and lysates of wild-type (WT) and ADAM10 KO MEF. Lack of shedding and no increased compensatory release of PrP is observed in ADAM10 KO cells. ADAM10 is shown in lysates as a proof of genotypes. Increased levels of PrPC are found in lysates and quantified by referring to β-actin (n = 8; p = 0.00005). (JPEG 131 kb) [file 13024_2018_248_MOESM8_ESM.jpg]

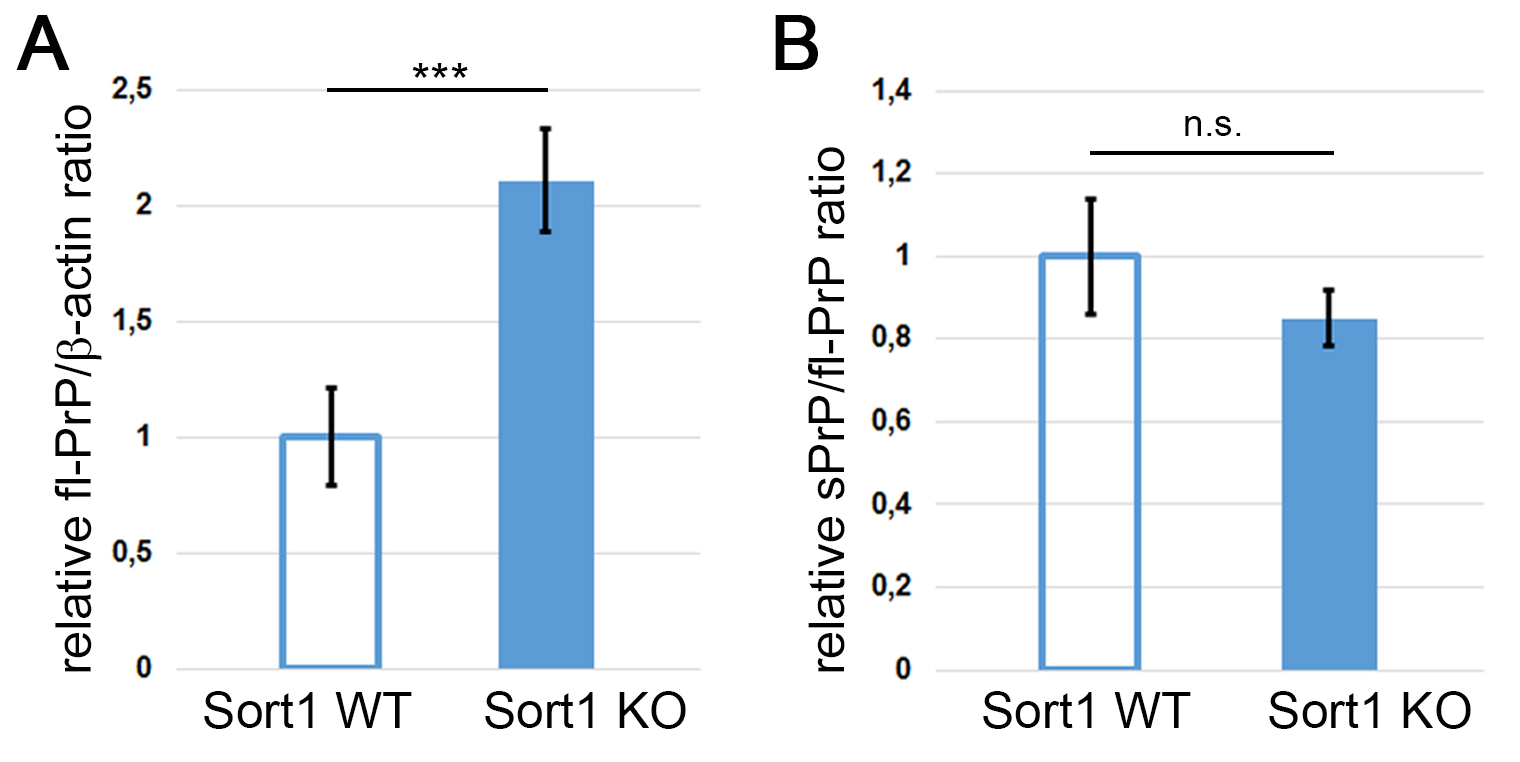

Supplement: Supplementary file 9 — (.jpg) Quantification of fl-PrP levels and ratio of sPrP/fl-PrP in Sort1 knockout mice. These quantifications refer to main Fig. 5j. (A) Increased amounts of fl-PrP are found in brains of Sort1 KO mice (2.11 ± 0.23; p = 0.0004; n = 4) compared to controls (WT set to 1.00 ± 0.21; SD). Actin served as loading control and for reference in densitometric quantification. (B) No significant differences are detected in the ratio of sPrP to fl-PrP between Sort1 KO mouse brains (0.85 ± 0.07, p = 0.128; n = 4) and controls (WT set to 1.00 ± 0.14). (JPEG 229 kb) [file 13024_2018_248_MOESM9_ESM.jpg]
